# A proposed workflow to robustly analyze bacterial transcripts in RNAseq data from extracellular vesicles

**DOI:** 10.3389/fmicb.2025.1486661

**Published:** 2025-03-20

**Authors:** Alex M. Ascensión, Miriam Gorostidi-Aicua, Ane Otaegui-Chivite, Ainhoa Alberro, Rocio del Carmen Bravo-Miana, Tamara Castillo-Trivino, Laura Moles, David Otaegui

**Affiliations:** ^1^Neuroimmunology Group, Biogipuzkoa Health Research Institute, P/ Doctor Begiristain s/n, Donostia-San Sebastián, Spain; ^2^Neurodegenerative Diseases Research Area of CIBER (CIBERNED), Carlos III Health Institute (ISCIII), Madrid, Spain

**Keywords:** extracellular vesicles, bacteria, RNA-seq, multiple sclerosis, taxa profiling

## Abstract

**Introduction:**

The microbiota has been unequivocally linked to various diseases, yet the mechanisms underlying these associations remain incompletely understood. One potential contributor to this relationship is the extracellular vesicles produced by bacteria (bEVs). However, the detection of these bEVs is challenging. Therefore, we propose a novel workflow to identify bacterial RNA present in circulating extracellular vesicles using Total EV RNA-seq data. As a proof of concept, we applied this workflow to a dataset from individuals with multiple sclerosis (MS).

**Methods:**

We analyzed total EV RNA-seq data from blood samples of healthy controls and individuals with MS, encompassing both the Relapsing-Remitting (RR) and Secondary Progressive (SP) phases of the disease. Our workflow incorporates multiple reference mapping steps against the host genome, followed by a consensus selection of bacterial genera based on various taxonomic profiling tools. This consensus approach utilizes a flagging system to exclude genera with low abundance across profilers. Additionally, we included EVs derived from two cultured species that serve as biological controls, as well as artificially generated reads from 60 species as a technical control, to validate the specificity of this workflow.

**Results:**

Our findings demonstrate that bacterial RNA can indeed be detected in total EV RNA-seq from blood samples, suggesting that this workflow can be a powerful tool for reanalyzing RNA-seq data from EV studies. Additionally, we identified promising bacterial candidates with differential expression between the RR and SP phases of MS.

**Discussion:**

This approach provides valuable insights into the potential role of bEVs in the microbiota-host communication. Finally, this approach is translatable to other experiments using total RNA, where the lack of a robust pipeline can lead to an increased false positive detection of microbial genera. The workflow and instructions on how to use it are available at the following repository: https://github.com/NanoNeuro/EV_taxprofiling.

## 1 Introduction

Extracellular vesicles (EVs) can be defined as membrane vesicles, naturally produced as lipid bilayer vesicles that contain a cargo (DNA, RNA, protein, lipids, etc.). The term has been used to refer to eukaryotic EVs and they have been widely studied in human blood demonstrating their huge importance in health and disease (Alberro et al., 2021). However, eukaryotic vesicles are not the only ones found in human biofluids. Bacterial EVs (bEVs) arise from the outer and inner membranes of gram-negative bacteria and cytoplasmic membranes of gram-positive bacteria through blebbing and lytic biogenesis pathways (Effah et al., 2024). Although bEVs from Gram-negative bacteria have been referred to as outer-membrane-vesicles (OMVs), bEVs could involve all the vesicles produced by bacteria (Hosseini-Giv et al., 2022). The presence of bEVs has been reported in human tissues such as saliva, feces and also in blood. Despite the potentially pathogenic component of these bEVs, they could also be a relevant communication tool between microbiota and the host organism (Tartaglia et al., 2018).

The influence of microbiota in several diseases is widely reported, including neurological and autoimmune diseases. Multiple sclerosis (MS) is a chronic, demyelinating, and immune mediated disease of the central nervous system (CNS) caused by a complex combination of genetic, epigenetic, and environmental factors. While variations in microbiome studies arise due to geographical, cultural, and dietary differences, there is consensus on certain microbial alterations in people with MS (pwMS), through several experiments in mice (Berer et al., 2017; Cekanaviciute et al., 2017) and through a huge collaborative effort that characterizes the microbiome in a large cohort of pwMS (Zhou et al., 2022).

These differences include a decrease in short-chain fatty acid (SCFA)-producing species and an increase in mucin-degrading species (Ghezzi et al., 2021). Broadly, MS is characterized by preserved microbial diversity with subtle alterations at the phylum level, notably an increased *Firmicutes*/*Bacteroidetes* ratio. However, more pronounced dysbiosis occurs at lower taxonomic levels, marked by increases in genera such as *Methanobrevibacter, Akkermansia, Acinetobacter, Pseudomonas, Blautia*, and *Ruminococcus*, including several mucin-degrading groups; and decreases in *Sutterella, Faecalibacterium, Prevotella, Fusobacterium, Anaerostipes, Clostridium* clusters XIVa and IV, *Parabacteroides*, and *Butyricimonas* (Ochoa-Repáraz et al., 2018; Ghezzi et al., 2021).

Recently, the international consortium for the study of the microbiome in MS (iMSMS) published findings highlighting a reduction in beneficial microorganisms associated with butyrate production, regulatory T-cell promotion, and inflammation attenuation. This reduction disrupts key metabolic pathways, potentially exacerbating inflammation and compromising the intestinal barrier (Zhou et al., 2022). Although most studies have focused on the gut microbiota in the relapsing-remitting (RR) form of MS, efforts are increasingly directed toward understanding its role in progressive forms of the disease. Progressive MS patients exhibit microbiota changes shared with RR patients when compared to healthy controls, including increased *Akkermansia* and *Clostridium*, and decreased *Dorea* and *Blautia* (Cox et al., 2021). The authors further identified, after adjusting for variables such as age, sex, race, ethnicity, and BMI, that *Enterobacteriaceae, Clostridium* g24 FCEY, and *Ruminococcaceae* FJ366134 were uniquely elevated in progressive MS. Their analysis concluded that disease status exerts the greatest influence on microbiota composition compared to other demographic factors.

Although traditional microbiota studies have primarily relied on sequencing the 16S rRNA region, recent approaches have emerged that integrate the analysis of RNA sequencing data. Several of these methods, along with their associated bioinformatic pipelines, have been described in prior studies (Bharti and Grimm, 2019; Calgaro et al., 2020; McClure et al., 2013). Most pipelines emphasize the importance of rigorous quality control (QC) and read filtering as highly relevant steps in the analysis (Bharti and Grimm, 2019).

Focusing specifically on EVs, Miceli et al. (2024) recently reviewed existing bioinformatic pipelines for the analysis of EV-derived RNA-seq data, complementing earlier research on EV characterization (Su et al., 2022; Saravanakumar et al., 2022). However, bioinformatic studies of EVs predominantly center on human EV characterization. Insights applicable to bacterial EVs are largely derived from adaptations of metagenomic analysis pipelines. Furthermore, most bioinformatics research in this area tends to prioritize the development of EV databases, species network analysis, or other downstream analyses, while the earlier stages of RNA-seq read processing and analysis remain relatively unexplored and offer significant opportunities for further improvement.

The primary aim of this study is to investigate the presence of bacterial transcripts as potential indicators of bacterial extracellular vesicles (bEVs) circulating in the blood of pwMS. To achieve this, we focus on analyzing how variations in sensitivity parameters and prior host genome mapping affect the detection of bacterial transcripts, among other fundamental tasks. These methodological adjustments aim to enhance the reliability and accuracy of bacterial read assignments, addressing potential sources of error from RNA-seq workflows. In addition to refining sensitivity and mapping strategies, this work also evaluates the use of multiple taxa profilers to analyze RNA-seq data. This integrative approach allows for a more robust evaluation of bacterial taxa and minimizes biases associated with relying on a single profiler.

## 2 Materials and methods

### 2.1 Sample and EV extraction

Whole blood was obtained from pwMS (10 RR and 10 SP) and age-matched healthy controls (*n* = 8) at the Donostia University Hospital (Iparraguirre et al., 2021) (Supplementary Table S1). Peripheral blood was collected by venipuncture into EDTA tubes and centrifuged at 1258g for 20 minutes.

EVs were isolated following a differential centrifugation step protocol as previously described by our group (Sáenz-Cuesta, 2015). Briefly, plasma aliquots were centrifuged at 13,000g for 2 min at 4°C. The supernatant was transferred to a new tube and centrifuged at 20,000g for 20 min at 4°C. The resulting pellet was resuspended with 100 μL DPBS (GIBCO, ThermoFisher, Waltham, MA, USA), which had been previously double-filtered through a 0.22 μm-pore filter to remove particles and aggregates, and was recentrifuged at 200,000 × g for 60 min. RNA was isolated using Trizol LS (ThermoFisher, Waltham, MA, USA) as previously described (Iparraguirre et al., 2021). RNA quantification was performed using a NanoDrop ND-1000 spectrophotometer (ThermoFisher, Waltham, MA, USA). The most frequent particle size ranges between 150 and 200 nm (Iparraguirre et al., 2021).

Samples were pooled (Supplementary Table S2) to achieve a minimum amount of 2 μg RNA. Following rRNA removal, libraries were prepared, and paired-end sequencing was carried out using Illumina HiSeq X Ten, obtaining an average of 40–50 × 10^6^ reads per sample.

### 2.2 Generation of control and *in silico* samples

To control for potential errors and biases in the analysis, both *in silico* and biological control samples were created. Biological control samples were generated by sequencing reads from EVs extracted from cultured *Lactobacillus acidophilus* and *Bifidobacterium lactis*. Notably, both species are commonly found in the human gut, making them suitable as positive controls.

*In silico* reads were generated from 61 species in varying proportions, including *Homo sapiens*, 25 bacterial species, 20 fungal species, and 15 viral species (detailed in Supplementary Table S3), using *InSilicoSeq* (v1.6.0) (Gourlé et al., 2018). Of the total 50 million reads, 40 million (80%) were assigned to *Homo sapiens*, while the remaining reads were distributed across the other species with relative abundances ranging from 0.1% to 0.625%. Some species were selected to belong to the same genera (e.g., *Blautia, Bacteroides, Aspergillus*, and *Rotavirus*) to evaluate potential profiling biases between species within the same genus and those across different genera.

The genomes used to generate the reads were downloaded from NCBI using the following command of BLAST suite (v0.3.3):

  ncbi-genome-download -F fasta -t {TAXID}

  --flat-output all

      -o {OUTPUT}/{TAXID} &&

  zcat {OUTPUT}/{TAXID}/*.fna.gz >

  {OUTPUT}/{TAXID}.fna

Reads were generated with the command:

  iss generate --genomes {TAXID}.fna --model

  hiseq --cpus 8

     --output {OUTPUT}/{TAXID}_reads  --n_reads

      {LENGTH_TAXID}

An individual FASTQ file was generated for each species. The reads were then sorted using the bash sort command, merged using cat, and shuffled with the bash shuf command. This approach ensured that no species was overrepresented in subsequent commands that utilized subsets of the FASTQ files to verify their integrity.

### 2.3 Generation of unified taxa profiler databases

To minimize profiler bias, a set of databases were generated that are based on the same set of organisms. A custom script was developed to create these databases, one for each profiler, following these steps:

(1) Protein FASTA and genome FASTA files were downloaded using ncbi-genome-download with the parameters -P -l complete,chromosome --flat-output for human, archaea, bacteria, protozoa, and viral taxa. For fungi, the parameter -l complete,chromosome,scaffold was applied. The following numbers of species were downloaded: archaea (628), bacteria (51,772), fungi (441), protozoa (53), and viral (14,977). Protein FASTA was used for *kaiju*, whereas genome FASTA was used for the rest of profilers.
(2) Individual protein and genome files were masked using *dustmasker* and *segmasker*, respectively, from the BLAST suite (v2.16.0).
(3) Databases were generated for each profiler. Additional details are provided in the source code (1A_build_profiler_dbs.sh).

Database generation and taxa profiling were performed on the Hyperion HPC at the Donostia International Physics Center, utilizing Intel Xeon Platinum 8362 and Intel Xeon Gold 6342 processors. Resources ranged from 4 to 32 CPUs and 256GB to 1,500GB of RAM, depending on the profiler.

### 2.4 Taxa profiling workflow

**Figure 1:**
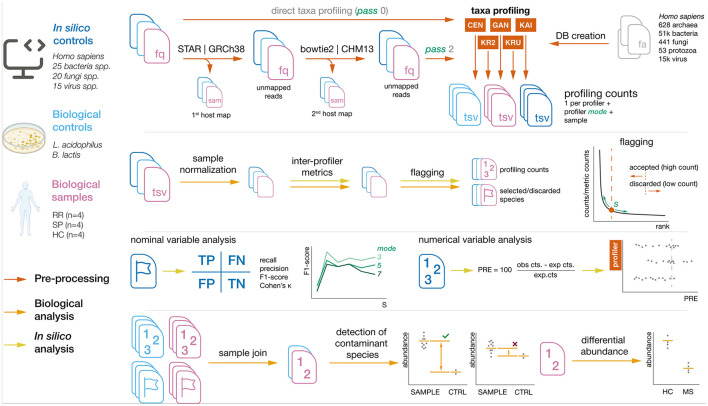
Summary of sample processing pipeline. Steps and sample origins are marked with their respective colors.

The processing workflow, depicted in Figure 1, consists of the following steps: (1) read mapping with to host reference with *pass0* and *pass2* strategies, (2) profiling using 9 different *modes*, (3) standardization of profiler output, (4) read count normalization, (5) profiler metric generation, (6) flagging, (7) inter-profiler data merging, and (8) filtering of species and genera.

#### 2.4.1 Read mapping to host reference

Reads from the samples were mapped to human reference genomes in two separate steps: first they were aligned to the GRCh38 genome and then to the more extensively sequenced CHM13 genome, as described by Gihawi et al. (2023).

The genomes used were downloaded via ncbi-genome-download (GRCh38 accession: GCF_000001405.40_GRCh38.p14; CHM13 accession: GCF_009914755.1_T2T-CHM13v2.0). Index generation for GRCh38 was performed with *STAR* (v2.7.10a) using the command STAR --runMode genomeGenerate with the additional parameter --genomeSAindexNbases 14. The salmon index was constructed using *salmon* (v1.10.2) with default parameters.

The first mapping was conducted using the *nf-core* rnaseq module (r version 3.14.0) with the GRCh38 genome. The following parameters were applied: --aligner star_salmon –skip_bbsplit --extra_salmon_quant_args “--minAssignedFrags 1” --save_unaligned--skip_qualimap --skip_pseudo_alignment.

The second mapping was carried out using *bowtie2* (v2.5.4) with the CHM13 genome. The index for CHM13 was built using default parameters, and reads were aligned with bowtie2 using the --very-sensitive argument.

In order to determine how host mapping affects the detection of non-host species by profilers, reads were profiled in two *passes*: *pass0* refers to the fact that reads were not mapped to the host, whereas in *pass2* reads were mapped to twice to the host, as mentioned in this section.

#### 2.4.2 Read profiling

Reads from plasma-derived EVs, biological controls, and *in silico* samples were mapped using the following profilers: *kaiju* (v1.10.1) (Menzel et al., 2016), *kraken2* (v2.1.3) (Wood et al., 2019), *krakenuniq* (v1.0.4) (Breitwieser et al., 2018), *centrifuge* (v1.0.4.2) (Kim et al., 2016), and *ganon* (v2.1.0) (Piro et al., 2020). Additionally, KMCP (v0.9.4) (Shen et al., 2022) was included but later discarded due to technical issues.

To account for variations in profiler sensitivity, nine unified *modes* (*mode* 1 to *mode* 9) were created, each with adjusted parameters specific to each profiler. Higher *mode* values correspond to a more lenient profiling, yielding a higher number of reads per genera or species, as well as more genera or species being discovered; whereas lower *mode* values result in a more stringent profiling.

For instance, in *mode* 1, *krakenuniq* used the parameter --hll-precision 18, while *kraken2* used --confidence 0.9. A detailed list of parameters is provided in Supplementary Table S4.

#### 2.4.3 Standardization of profiler output

Profiler outputs were standardized using *taxpasta* (v0.7.0) with the command: taxpasta standardize --add-name --add-lineage.

Standardization was performed in two levels: for biological samples the standardization was performed at genus level, whereas for *in silico* samples it was performed at species level. For that, the flags --summarize-at genus and --summarize-at species were used.

The reason for the two-level standardization is that one of the objectives within the analysis of *in silico* samples is the evaluation of the accuracy of within-genera species detection, whereas for biological samples we follow standard practice of reporting results at a genus level.

The profiling output consisted of a table detailing the number of species or genera and their respective read counts for each profiler. One table is obtained for each combination of profiler and *mode*.

Then, in order to obtain one merged table across profilers, read counts from individual profilers were concatenated using an outer join to consider all possible species. The read counts (*R*_*ij*_) were defined as the vector of counts assigned to all species shared across profilers after the join:


$$R_{ij}=\left(r_{ij1},r_{ij2},\ldots ,r_{ijn}\right),\;n\in \text{species}$$

where *r*_*ijk*_ represents the count of reads assigned to species *k* by profiler *j* in sample *i*.

Species with zero counts across all profilers were discarded.

#### 2.4.4 Read count normalization

To account for differences in sequencing depth, raw read counts (*R*_*ij*_) for each profiler in sample *i* were normalized using a correction factor (*CF*) based on the ratio of the median FASTQ length across all samples (MedianLength) to the FASTQ length of sample *i* (*L*_*i*_):


$$CF_{i}=\frac{\text{MedianLength}}{L_{i}}$$

Normalized counts ($R_{ij}^{\text{norm}}$) for profiler *j* in sample *i* were calculated as:


$$R_{ij}^{\text{norm}}=R_{ij}\cdot CF_{i}$$

This normalization step is skipped for *in silico* data because only one sample was generated.

For biological samples, considering that biological samples and controls were sequenced at different depths (approximately 50M reads per sample and 2.5M reads per control) two types of normalization were performed. The first normalization method is a joint normalization of samples and controls jointly, whereas in the second normalization samples and controls are normalized separately.

#### 2.4.5 Profiler metric generation

After normalization, the weighted average, standard deviation (SD), and coefficient of variation (CV) were calculated for both raw and normalized counts. The weighted average (μ) for each sample was defined as:


$$\mu_{j}=\frac{\sum_{j\in \text{profilers}}R_{ij}\cdot w_{j}}{\sum_{j\in \text{profilers}}w_{j}}$$

where *w*_*j*_ is the weight associated with profiler *j*, calculated as:


$$w_{j}=\frac{1}{{\text{TotalCounts}}_{j}}$$

The weighted standard deviation (σ) was computed as:


$$\sigma_{j}=\sqrt{\frac{\sum_{j\in \text{profilers}}w_{j}{\left(R_{ij}-\mu \right)}^{2}}{\sum_{j\in \text{profilers}}w_{j}}}$$

The coefficient of variation (CV) was calculated as:


$${\text{CV}}_{j}=\frac{\sigma }{\mu }$$

Therefore, for each sample a set of metrics is generated for raw counts ($\mu_{j}^{raw}$, $\sigma_{j}^{raw}$, ${\text{CV}}_{j}^{raw}$) and normalized counts ($\mu_{j}^{norm}$, $\sigma_{j}^{norm}$, ${\text{CV}}_{j}^{norm}$).

#### 2.4.6 Flagging

In the flagging step, species or genera in each sample are marked (flagged) as “accepted” or “rejected” based on theis values in the metric columns (counts of each profiler and associated statistics, both raw and normalized). In this step, species or genera were sorted by value, and the kneedle algorithm, as implemented in the *kneed* package (v0.8.5) (Satopaa et al., 2011), was applied to determine a cut-off value. Species or genera with values below this cut-off were flagged as rejected.

The kneedle algorithm is a curve point detection method that identifies the point of maximum curvature on a rank distribution. This approach is effective when the distribution of reads counts–or other values–, sorted by rank, exhibits a “hockey stick” shape, where a small number of read counts have significantly higher values compared to the rest. Species or genera falling in the lower end of the curve can be interpreted as unlikely to be present in the sample, that is, they are likely generated as a *byproduct* during the profiling step; or can also be interpreted as potential contaminants with a low abundance in the sample.

The kneedle algorithm incorporates a hyperparameter, *S*, which adjusts the curve point. Higher *S* values lower the cut-off threshold, resulting in more species or genera being flagged as accepted. To account for variability, an array of *S* values (0, 1, 2, 3, 4, 5, 6, 7, 10, and 15) was used. These values were arbitrarily chosen, reflecting a range between a conservative flagging (*S* = 0) to a very lenient flagging (*S* = 15).

The output of the flagging process include (1) a counts table containing raw and normalized counts per profiler, along with their weighted mean, standard deviation (SD), and coefficient of variation (CV) across profilers; and (2) a boolean flag table with the same dimensions as the counts table, indicating species selected based on the curve point detection criteria.

#### 2.4.7 Inter-profiler data merging

After processing individual samples, a merged table was created by combining the normalized counts of weighted averages for each sample (12 plasma-derived EV samples and 2 biological controls), using an outer join, to retain species that were not captured in all sample. Empty values were filled with NaN instead of 0 to differentiate non-existing genera from low-abundance genera.

This step was not applied to *in silico* data, because only one sample was generated.

#### 2.4.8 Filtering of species and genera

Similarly to the previous step, this step was only performed to the merged table of biological samples and controls.

The merged weighted averages table was subjected to three additional filtering steps.

In the first filtering step, genera present in less than 65% of samples were discarded. This value was set to ensure that genera belonging to at least two of three scenarios (HC, SP or RR) were retained, and therefore can be adjusted base on the specific attributes of each dataset.

The second filtering step assessed the number of flags assigned to each genus across samples. For each genus, the number of samples passing the cut-off criteria defined in the *Flagging* section was computed using the flag table. A cumulative sum curve of species versus the number of flags was generated, resulting in a ranked distribution. The kneedle algorithm was applied to this curve to determine the minimum number of flags required for a genus to be retained. For this step an *S*_flag_ value of 0 was set–default value–, although it can be adapted based on the attributes of the dataset. Genera meeting this threshold were selected for further analysis.

The aim of these first two steps is to retain species with considerable abundances that are stable across samples, in order to reduce false positive bias, that is, to avoid reporting on species that are exclusive of one type of sample, which may be a product of sample processing.

The third step is applied to filter genera that have similar abundance level in controls and in samples, which are therefore assumed to be contaminant. This step also uses the kneedle algorithm. For each genus, the median number of normalized weighted average counts across samples and the maximum number of normalized counts in controls were calculated. A threshold (*T*) was defined such that


median(samples)>T·max(control)

For a threshold range of *T* ∈ [1, 500], the proportion of species discarded at each threshold was computed, and the kneedle algorithm was applied to the rank distribution. Similar to the previous step, threshold value *T* is dependent on the S value assigned at this step (*S*_diff_ = 0), which can also be adapted.

### 2.5 Analysis of *in silico* species

The aim of this analysis was to evaluate the performance of different profilers and assess the effects of (1) profiling with or without prior host mapping, (2) the sensitivity modes used during profiling, and (3) the *S* parameter applied during the flagging step. Our goal was not to determine the optimal set of parameters but rather to gain a general understanding of how each parameter influences the workflow and to identify its underlying limitations. This knowledge can then be applied to the analysis of biological samples.

To achieve this, the *in silico* dataset, which includes 60 “ground truth” species, was used to create two types of variables: nominal variables derived from the presence/absence of a species and numerical variables derived from the counts assigned to the original species.

### 2.6 Comparison of detected species between *pass0* and *pass2*

To understand how prior host mapping affects the detection of non-human species, the Jaccard index between the species detected in *pass0* and *pass2* is computed. If *P*_0_ is the set of species detected in *pass0* and *P*_2_ is the set of species detected in *pass2*, the Jaccard index is computed as:


$$J=\frac{P_{0}\cap P_{2}}{P_{0}\cup P_{2}}$$

#### 2.6.1 Nominal variable analysis

For nominal variables, the number of true positives (TP), false positives (FP), false negatives (FN) and true negatives (TN) is computed, based on the presence or absence of counts to species originally present in the list of 60 species described in Supplementary Table S3.

Then, precision ($\frac{TP}{TP+FP}$), recall ($\frac{TP}{TP+FN}$), F1-score ($2\frac{\text{precision}\cdot \text{recall}}{\text{precision}+\text{recall}}$), and Cohen's kappa (κ) were computed. Cohen's kappa was defined as:


$$\kappa =\frac{p_{0}-p_{e}}{1-p_{e}},\;p_{0}=\frac{TP+TN}{N},\;N=TP+FP+FN+TN$$

$$p_{e}=\left(\frac{TP+FP}{N}\cdot \frac{TP+FN}{N}\right)+\left(\frac{TN+FP}{N}\cdot \frac{TN+FN}{N}\right)$$

To evaluate A/B comparisons (e.g., profiling with or without prior host mapping), the χ^2^ statistic was calculated from the contingency table:


$$\left(\begin{array}{cccc} TP_{A} & FP_{A} & FN_{A} & TN_{A}\\ TP_{B} & FP_{B} & FN_{B} & TN_{B} \end{array}\right)$$

A significance level of α = 0.1 was used to determine statistically significant differences in the contingency table.

#### 2.6.2 Numerical variable analysis

Numerical value analysis is performed with the species originally present in Supplementary Table S3, where the number of observed and expected counts are compared. To that end, the percent relative error (PRE) and percent absolute error (PAE) were calculated, along with their means (MRE and MAE) and standard deviations (MRED and MAED):


$$PRE_{i}=100\cdot \frac{x_{obs,i}-x_{exp,i}}{x_{exp,i}},\;PAE_{i}=100\cdot \frac{\left|x_{obs,i}-x_{exp,i}\right|}{x_{exp,i}}$$

$$MRE=E\left[PRE_{i}\right]=\frac{100}{N}\sum \frac{x_{obs,i}-x_{exp,i}}{x_{exp,i}},\;MRED=\sigma \left(PRE_{i}\right)$$

$$MAE=E\left[PAE_{i}\right]=\frac{100}{N}\sum \frac{\left|x_{obs,i}-x_{exp,i}\right|}{x_{exp,i}},\;MAED=\sigma \left(PAE_{i}\right)$$

### 2.7 Analysis of biological samples

The analysis of biological samples consists of two main aspects: the effect of the detection of contaminant genera and the analysis of differentially abundant genera across conditions.

#### 2.7.1 Detection of contaminant genera

In the section “Read count normalization” of the materials and methods, two normalization approaches were applied to normalize counts between biological samples and controls: a joint normalization including samples and controls (method A) and a separate normalization (method B).

Based on these normalization strategies, genera were categorized into three groups: (1) genera discarded using both normalization methods, which are contaminants with a high presence in control samples; (2) genera discarded with method A but retained with method B, which are contaminants with a lower abundance, which after normalization is comparable to the abundances of biological samples; and (3) genera retained by both methods, which have very low abundance in controls and thus are likely to be present in biological samples.

#### 2.7.2 Analysis of differentially abundant genera

Once the genera passing the normalization threshold were selected, their abundance across samples were compared. Comparisons were performed between the following groups: (1) HC and RR, (2) HC and SP, (3) RR and SP, and (4) male and female samples. Statistical significance was evaluated using the Mann-Whitney U test, with multiple comparisons corrected using the Benjamini-Hochberg procedure.

Additionally, due to effect of low number of samples in the statistical analysis, genera with *p*-value < 0.15 were sorted by absolute log_2_ fold change and reported.

## 3 Results

### 3.1 Profiler-specific differences in host read assignment and filtering efficiency

To evaluate the performance of different profilers, the proposed methodology includes the analysis of three types of samples: plasma-derived EVs from study subjects, two biological controls pooled from cultured bacteria-derived EVs, and an artificially generated dataset combining reads from 60 species, including human reads.

Taxa profiling studies often face challenges in assigning reads to the correct taxa due to contaminating reads–arising from the host species or sequencing kits–being incorrectly assigned to other species, leading to high rates of false positives (Gihawi et al., 2023). To mitigate this issue, the pipeline incorporates two critical steps: a three-fold mapping to the human genome and a flagging system.

The three-fold mapping approach involves sequentially mapping reads against the host genome three times (twice to the host genome using two different aligners and a third time during profiling). Following the first mapping, a median of approximately 9.5% of reads remains unmapped (Supplementary Table S5). This percentage decreases to 7% after the second mapping. However, the third mapping, performed by the profilers themselves, still assigns 1.5% of reads to humans. Notably, the median percentage of reads mapped to humans varies depending on the profiling mode, with 1.24% assigned at mode 3 and 1.92% at mode 7.

Regarding biological controls, we observe that 1%–4% of reads are assigned to the human genome during the initial mapping. However, unlike other samples, subsequent mappings to the genome in the second pass or by profilers are nearly zero.

The proportion of reads assigned to human and non-human species differs substantially between profilers. For instance, *kaiju* and *kraken2* exhibit the lowest human read mapping rates, with median ranges, calculated across biological samples, for *kaiju*, of 0.52% (minimum–maximum: 0.27–1.19) for *mode* 3 and 0.93% (0.55–1.93) *mode* 7 respectively; and, for *kraken2*, of 0.12% (0.07–0.24) for *mode* 3 and 0.73% (0.39–1.12) *mode* 7 respectively.

In contrast, *centrifuge* and *ganon* demonstrate higher mapping rates. For *centrifuge*, it ranges between 1.82% (1.02–3.55) for *mode*3 and 2.92% (1.41–4.71) for *mode*7; and for *ganon* the ranges are between 1.29% (0.63–1.62) for *mode*3 and for 2.07% (1.05–2.87), respectively. These differences suggest that each profiler exhibits specific patterns affecting read detection, which will be further examined in subsequent analyses.

### 3.2 Analysis of profiler performance in *in silico* samples

#### 3.2.1 Prior host mapping offers a slight improve in profiling

**Figure 2:**
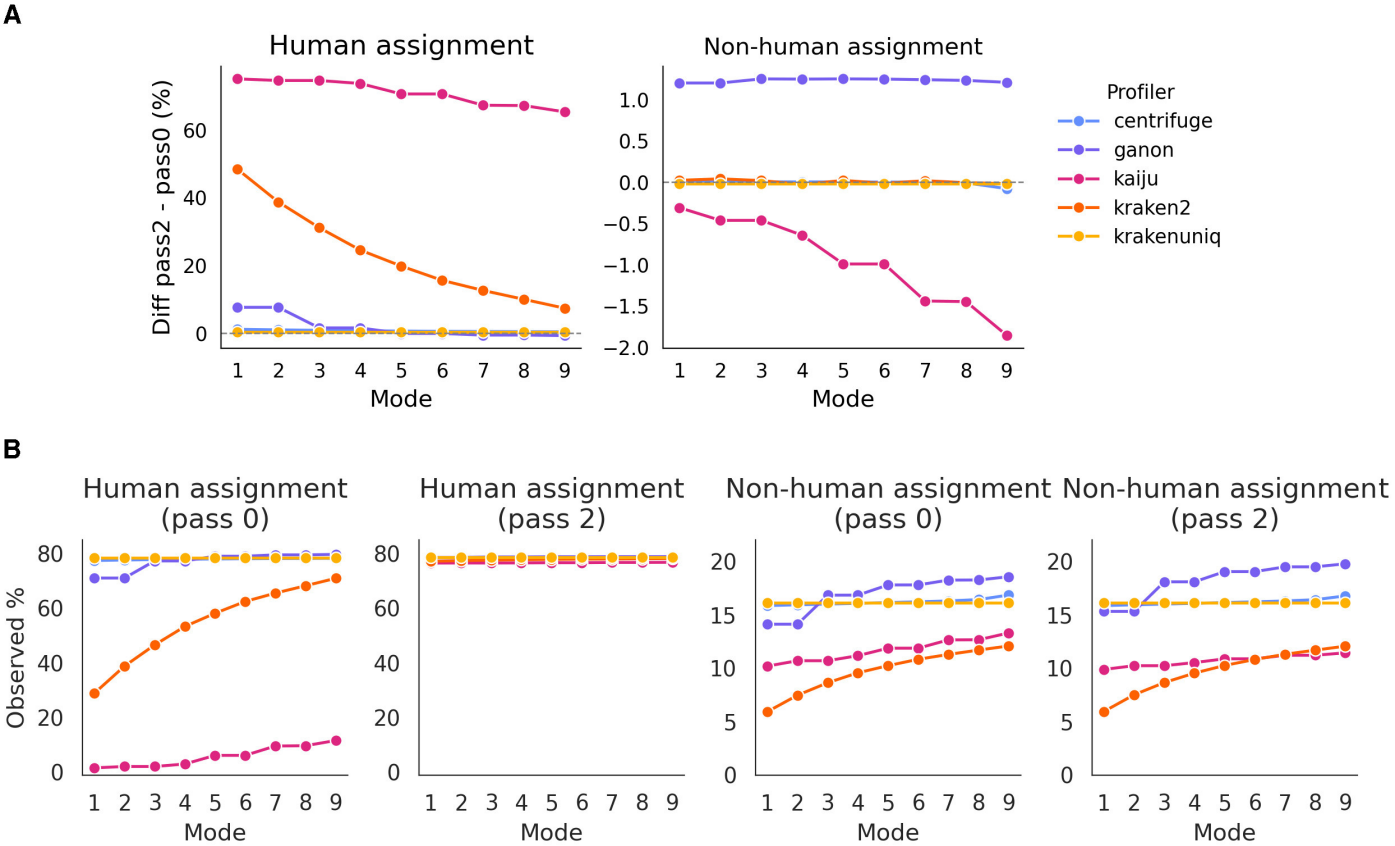
**(A)** Difference in assignment to human and non-human species across different profilers between *pass2* and *pass0*. The figure illustrates the variability in the number of reads assigned, highlighting the reduction in false positives when using *pass2*. Values near 0 indicate that the profiler read detection was the same as the expected number. **(B)** Percentage of observed counts assigned to human and non-human species, with *pass0* and *pass2* settings.

One of the aspects contested by Gihawi et al. (2023) is that the lack of prior mapping to the host genome is crucial to minimize false positive biases in the assignment of species. To evaluate this, we performed taxa profiling under two conditions: direct profiling without prior host mapping (*pass0*) and profiling after reads were mapped twice to the host genome (*pass2*). We observed that while the number of reads assigned to humans varied significantly between profilers, particularly with *kaiju* and *kraken2*, which showed a reduced mapping to human, there were no substantial differences in the assignment to non-human species, especially for *krakenuniq, kraken2*, and *centrifuge* (Figure 2). For these cases, the number of assigned reads was almost identical between *pass2* and *pass0*.

Interestingly, this effect appears to be independent of the profiling *mode* for non-human species, although a slight decrease in assignment was noted for *kaiju*. Conversely, for human species, the differences decreased as expected; since higher profiling *modes* are less stringent, increasing the likelihood of mapping to human species. Additionally, the Jaccard index for the total number of detected species remained consistently above 0.9 across all profilers and *modes* (Supplementary Figure S1). The variations in the Jaccard index depended on the profiler and mode, without a clear pattern. Expectedly, the number of detected species increased with the *mode*, for most profilers: linearly for *kaiju* and *kraken2*, and exponentially for *centrifuge* and *ganon*. Interestingly, *krakenuniq* reported the same number of detected species regardless of mode.

The total number of reported species, regardless of detection dynamics based on *mode*, is remarkably high–exceeding 1000 in most profiler/*mode* combinations–despite the fact that only 60 non-human species should be present. This suggests that a substantial portion of these species should be flagged as absent or negative after processing. The *S* parameter plays a key role in this filtering process, with higher values leading to more species being classified as present in the sample.

To better understand how the *pass* may affect the filtering of species, we computed the truth table (TP, FN, FP, and TN), Cohen's κ, and the χ^2^ statistic for the contingency table across 540 profiler, *mode*, and *S* parameter combinations (6 profilers–5 profilers + the weighted mean values across profilers– × 9 *modes* × 10 *S* values). Of these 540 combinations, only 38 (7.03%) showed statistically significant differences between *pass0* and *pass2* (α = 0.1). When looking at what properties made these combinations to be different, we observed a clear bias toward specific *S* values: of the 37 combinations, 12 where for *S* = 0 and 10 for *S* = 3. There was also a profiler bias: *ganon* was present in 13 cases, and the weighted mean in 15 cases. Lastly, mode values were equally distributed, and thus there was no apparent mode dependency.

As a side note, regarding the relationship between the F1-score and κ value, we observed a nearly identical correlation between both measures (Supplementary Figure S2). Given the way both formulas are constructed, they reward correct classifications (high TP and TN rates) while penalizing errors (FP and FN). Consequently, a strong correlation between these measures indicates a high proportion of TP and TN and a low proportion of FP and FN. Considering this redundancy, we have chosen to use the F1-score as the primary performance metric.

**Figure 3:**
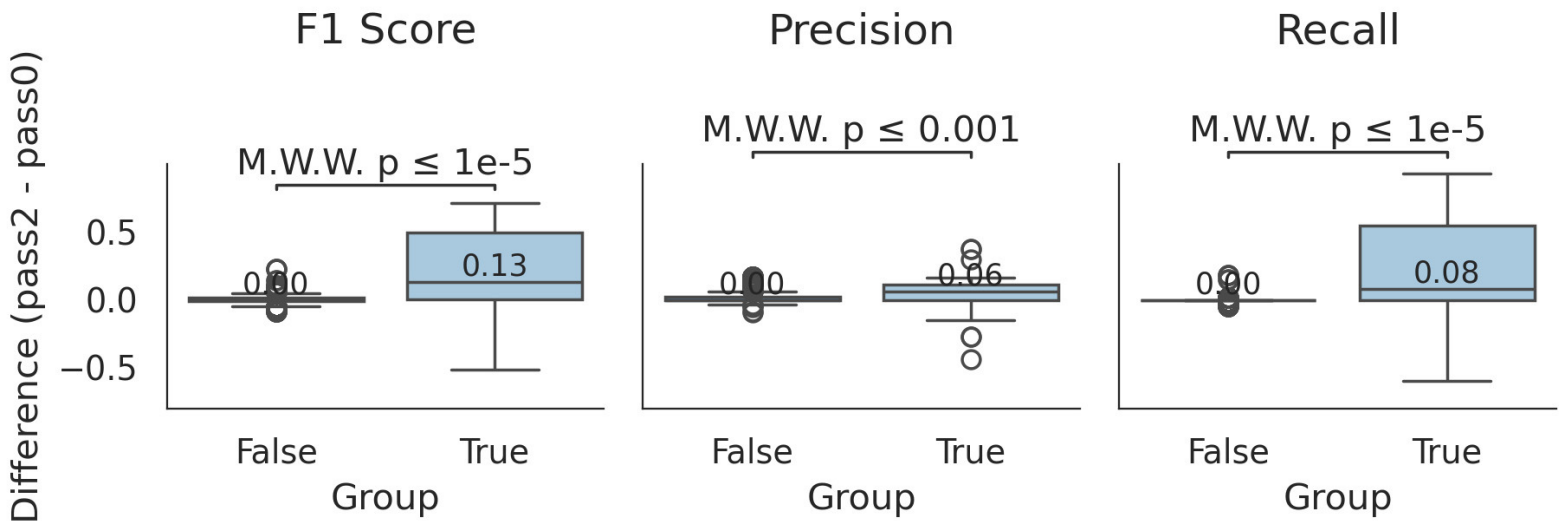
Differences between *pass0* and *pass2* profiling. Boxplots indicating the difference in F1-score, precision and recall for *mode, S* and profiler combinations that were/were not statistically significant at the χ^2^ test (group True/False). Values higher than 0 indicate an improvement in the respective metric of *pass2* against *pass0*. Median values of the boxplot distributions are indicated, as well as the Mann-Whitney U test *p*-value between the cases of the two conditions.

Interestingly, in cases where the χ^2^ difference was statistically significant, slight improvements were observed in precision, recall, and F1-scores (Figure 3). The median differences showed 0.06 increase in precision, 0.08 in recall, and 0.13 in F1-score (Mann-Whitney U test, *p* ≤ 0.001 for all comparisons). Notably, for specific profilers and *S* values, median recall and F1-score increased drastically (e.g., by 0.57 and 0.54 for *S* = 3 and by 0.55 and 0.52 for *ganon*). Overall, *pass2* outperformed *pass0*, particularly in increasing TP values and decreasing FN values.

Focusing on the selected 60 species that constitute the *in silico* dataset, we found a near-perfect correlation between *pass0* and *pass2* observed counts (Supplementary Figure S3A). The correlation between expected and observed counts was similarly high, with slight improvements for *pass2* (Supplementary Figures S3B, C). For instance, the linear regression slope for *pass2* vs. expected counts was 0.72, compared to 0.70 for *pass0*.

Thus, while prior host mapping may yield negligible differences for detecting non-human reads, *pass2* still offers slight improvements, particularly in reducing false negatives, and is recommended for better performance.

#### 3.2.2 Intermediate *S* values show best performance for species detection

**Figure 4:**
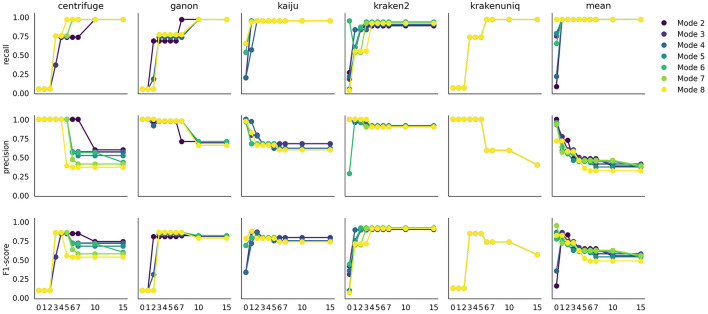
Effect of the *S* parameter on recall, precision, and F1-score. Each plot represents the evaluation metric at a specific *S* value, with different *mode* values in different colors.

During the flagging step, species with low counts are flagged as not selected. This process depends on the distribution of counts for a given profiler and mode and is automatically determined using a knee point detection method. To select more or fewer species, the *S* parameter can be adjusted. To understand how species detection is affected by this parameter, Figure 4 illustrates the recall, precision, and F1-scores across a range of *S* values and profilers.

Each profiler exhibits a specific pattern, influenced by the profiling mode, but two major trends are consistently observed. First, recall increases with higher *S* values, typically within a range of 1 to 7, depending on the profiler. Conversely, precision decreases as *S* increases. This trade-off is expected: a higher *S* value includes more species, capturing most true positives (higher recall) at the cost of introducing more false positives (lower precision). The combined effect is reflected in the F1-score, which peaks at *S* values between 2 and 5. Interestingly, most profilers show a plateau in the F1-score after a certain *S* value, indicating that the knee point detection algorithm effectively avoids including additional species that would excessively increase the false positive ratio.

Using the weighted average across profilers rather than individual profiler outputs offers an advantage, providing better stability in precision across *S* values and modes. Each profiler has a unique dynamic, but the averaged value smooths these differences. Based on these findings, we recommend avoiding extremely low (*S* = 0) or extremely high (*S* = 10 or 15) values, as they can skew the detection FN or FP species.

#### 3.2.3 Higher *mode*s result in a slightly improved detection of species

During profiling, each profiler has specific parameters to control their sensitivity. The *mode* parameter is a set of combinatory values applied to the profilers. Higher *mode* leads to less stringent read assignment. To understand how *mode* impacts species detection, Supplementary Figure S4 presents recall, precision, and F1-scores for different *mode* values.

Based on the truth table, precision, recall, and F1-scores are more *S*-dependent and profiler-dependent than *mode*-dependent. As previously mentioned, higher *S* values increase recall but reduce precision, and vice-versa. However, higher *mode* values only decrease the precision slightly—reflecting the inclusion of new species and an increased number of false positives—and do not affect the recall. This effect is even the oposite for extreme *S* values (0, 15) for specific profilers; highlighting the effect of filtering of species over the choice of *mode*.

When analyzing the number of counts detected for existing species from the *in silico* dataset, increasing the *mode* results in a modest improvement in the number of detected reads, as shown in Supplementary Figure S5. The correlation of counts, reported as the R^2^ Pearson correlation coefficient and the slope of the linear regression between expected and observed counts, increases slightly. Similar trends are observed in reductions of mean absolute error (MAE). Interestingly, mean absolute error deviation (MAED) shows profiler-specific behavior, that is, an increase or decrease depending on the profiler (Supplementary Figure S5B). For weighted average counts, MAED consistently decreases at higher *mode* values. This trend may be expected, as additional reads either remain unassigned to expected species or are properly assigned, thereby improving these metrics.

In summary, despite the increase in species introduced by higher *mode* values, as shown in Supplementary Figure S1, the precision remains largely stable across *mode*s, demonstrating that the flagging of non-accepted species using the knee method is highly effective. Therefore, *mode* values are not as critical as initially thought. However, considering the slight accuracy increase in the number of reads detected, we recommend using *mode* values between 5 and 7.

#### 3.2.4 Weighted average counts reduce variability in read detection across profilers

**Figure 5:**
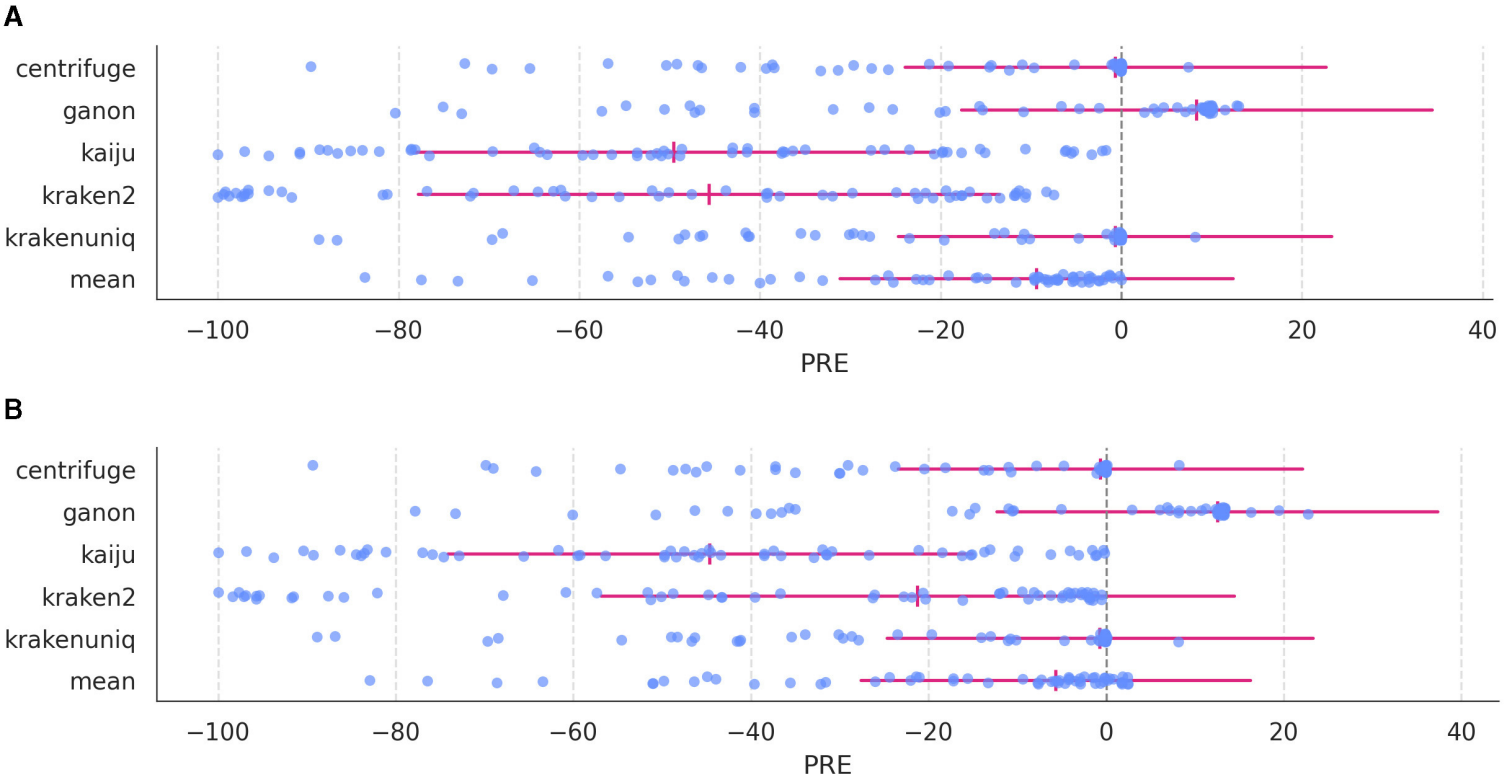
Percent relative error (PRE) for each species in the *in silico* dataset across profilers. **(A)** PRE distribution at *mode* 3; **(B)** PRE distribution at *mode* 7. The expected zero value is marked with a gray dashed line, and for each profiler the MRE (vertical red line) and MRED (horizonal red line) are indicated.

Having analyzed the effects of *mode, S*, and *pass* on detecting true and false species and read counts, we explored biases in read detection across profilers. Figure 5 presents the percent relative error (PRE) for each species in the *in silico* dataset.

Each profiler exhibited distinct PRE distributions. For instance, *kaiju* and *kraken2* showed the lowest PRE values, while *centrifuge* and *krakenuniq* displayed better distributions, with many species near zero (identical expected and observed counts). Conversely, *ganon* exhibited slightly elevated PRE values for most species, along with some negative values. The weighted average combines these results into a single PRE set that reduces the biases of individual profilers, resulting in values slightly below zero for most species. While the weighted average counts do not yield the lowest MRE values, it is comparable to top-performing profilers such as *centrifuge, ganon*, and *krakenuniq*.

Interestingly, as shown in Supplementary Figures S5B, C, MAE and MRE values approach zero modestly with higher *mode* values for all profilers as well as with the weighted average counts. However, the most significant difference occurs in MRED values, for which the values associated to weighted average counts remain the smallest across profilers (Wilcoxon rank-sum test, FDR correction, *W* = 45, *p* = 0.002 for all MRED value comparisons between the weighted average counts and other profiler) (Supplementary Figure S6).

**Figure 6:**
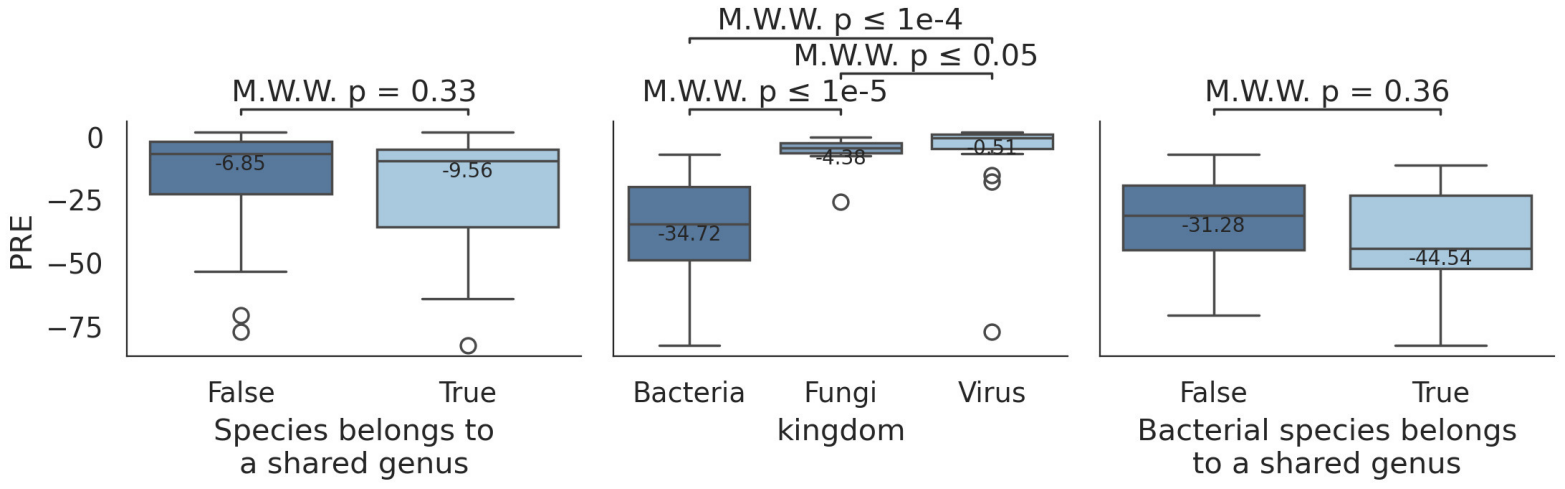
Differences in PRE across species and genera under *mode* 5, using weighted average counts. Left boxplot indicates PRE values for species that belong to a shared genus (True) vs. those who do not (False). Right plot shows the same information, but restricted to bacterial species. Mid box plot represents PRE values for *Bacteria, Fungi*, and *Virus*.

Analysis of PRE values (Figure 5) reveals that some species consistently show close-to-zero PRE values, while others exhibit extreme deviations, between -40 and -80. Initially, this was thought to stem from species sharing the same genus, but PRE values of species belonging to the same genus are similar to those from deferent genera (two-sided Mann-Whitney U test, *U* = 440, *p* = 0.33) (Figure 6). However, difference in PRE values across kingdoms (*Bacteria, Fungi*, and *Virus*) were evident: *Bacteria*-related species consistently showed lower (worse) PRE values compared to *Fungi* and *Virus* (two-sided Mann-Whitney U tests, fungi: *U* = 12, *p* = 1.18·10^−7^; bacteria: *U* = 31, *p* = 2.54·10^−5^).

Comparison of PRE values between bacterial species that share genus against species that do not share them still show no significant differences (two-sided Mann-Whitney U test, *U* = 78, *p* = 0.36). This tendency is shared across profilers (Supplementary Figure S7) and modes (Supplementary Figure S8). Therefore, a worse PRE values does not stem from two similar species being profiled incorrectly; rather, other factor are influencing this effect.

The differences in PRE values may stem from the significant variation in the number of species recorded for each kingdom in the database. Bacteria, with 51,772 species, have a much larger representation compared to fungi (441) and viruses (14,997). This disparity increases the likelihood of reads being assigned to similar taxa within bacteria, which could explain the greater variability in PRE values observed for this kingdom. However, the amount of viruses is in the same order of magnitude than that of bacteria, and their PRE values are near to zero. Therefore, this effect cannot neither entirely account for the differences in low PRE values assigned to bacterial species.

To summarize, each profiler shows highly specific detection patterns that are not much influenced by *mode*. Additionally, although the use of weighted averages may only slightly improve the amount of detected number of reads, it reduces the variation of reads across species, providing more consistent results.

These findings suggest that other factors, such as genome structure, sequence similarity, or taxonomic diversity, may contribute to the observed patterns. Understanding these additional influences could provide a clearer explanation for the differences in PRE values across kingdoms.

### 3.3 Analysis of profiler performance in biological samples and controls

After analyzing the effects of parameters on the detection of species in the *in silico* dataset, we proceed to analyze biological samples and controls to identify genera of potential interest.

#### 3.3.1 Effect of *S* and *mode* is consistent across biological samples and controls

The first aspect examined is the impact of *mode* and *S* on the detection of genera and how many of the detected genera are later retained after filtering steps.

The total number of detected genera remains relatively stable across different *mode* values, with an increase of 4%–11% in detected genera between *mode* 3 and *mode* 7 across different samples (Supplementary Table S6). This effect is more pronounced in the biological controls, showing an 18% increase for ACIDOLA and 55% for BLACTIS, despite these controls detecting fewer genera overall compared to other samples. This discrepancy may be due to a higher baseline diversity of genera in the controls, many of which are likely contaminants or false positives, as more than 99% of genera detected in *mode* 3 are also present in *mode* 7, indicating that higher *mode* values generally introduce additional genera.

**Figure 7:**
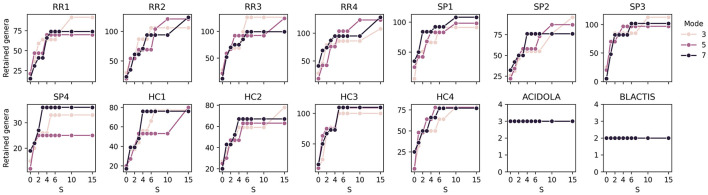
Effect of *S* values on the number of retained genera. Each subplot corresponds to a specific sample, and each line represents a different *mode* value.

Focusing on the numbers of retained genera, (Figure 7, Supplementary Figure S9) illustrate the number of retained genera and the corresponding retention percentages, respectively. The number of retained genera remains stable, ranging between 80 and 100, which corresponds to approximately 4–6% of the total detected genera. Analyzing the control samples, ACIDOLA consistently retains three genera (out of 563 and 668 with modes 3 and 7): *Lactobacillum, Leifsonia*, and *Salmonella*, with 56M, 245k, and 94k reads, respectively. Similarly, BLACTIS detects two genera, *Bifidobacterium* and *Staphylococcus*, with 60M and 11k reads, respectively. These results suggest that the detection is robust and largely unaffected by variations in *S*, while introducing only a minimal number of false positives. This robustness implies that the detection process in other samples may also exhibit a similar tendency toward a limited introduction of false positives.

Regarding the detection of genera in biological samples, an expected trend emerges with increasing *S* values, where the number of detected genera rises. However, this increase stabilizes when *S* reaches values of approximately 5–7, consistent with findings from the *in silico* sample analysis. This stabilization indicates that higher *S* values do not necessarily contribute to the detection of additional genera once a plateau is reached, and therefore all the additionally detected genera are likely to have extremely low read counts and thus be contaminants or artifacts.

The influence of *mode* on genus detection is generally minimal, also consistent with findings *in silico*, with the exception of samples SP4 and HC1, where the detection is similar for *mode* 3 and 7, while it decreases for *mode* 5. However, counterintuitively, in some cases, slightly more genera are detected with *mode* 3 than with less stringent modes. This phenomenon might be explained by a less steep ranking curve of genera based on their counts when fewer genera are detected. In such cases, the detection algorithm may become “more permissive", setting the cutoff at a higher threshold. Despite these anomalies, the results confirm that the algorithm is robust and capable of producing consistent outcomes across a reasonable range of *S* values and *mode* settings.

Therefore, based on the conclusion arrived at this section, the following analyses will be performed using *mode* 3 and *S* 7.

#### 3.3.2 Similarities between profilers reveal profiler-specific patterns

**Figure 8:**
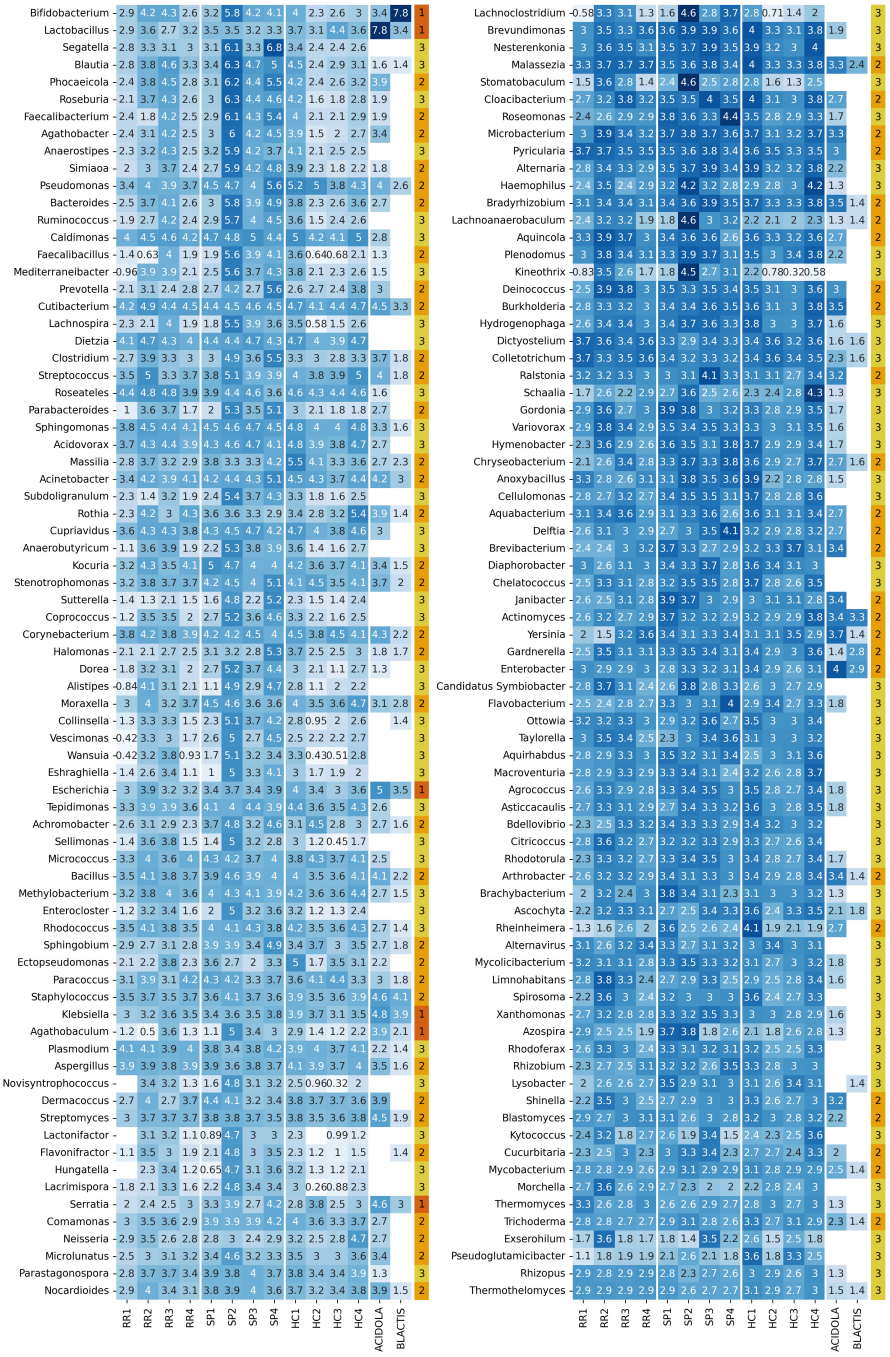
Heatmap with log_10_ abundance of retained genera. The last column indicates the contaminant status: (1) genera discarded after separate and joint normalization (contaminants with a high presence in control samples), (2) genera discarded after joint normalization, but kept with separate normalization (contaminants with a lower abundance), and (3) genera retained after normalization (likely to not be contaminants).

The resulting set of genera based on the selected *S* and *mode* parameters consists of 151 genera, as shown in Figure 8.

The distribution of counts assigned to each genus (Supplementary Figure S10A) reveals a consistent pattern across all samples, with two distinct groups of profilers: *centrifuge, ganon*, and *krakenuniq*, along with the weighted average, report higher read counts, while *kaiju* and *kraken2* show lower counts. This distribution is comparable to the PRE distribution observed in the *in silico* dataset (Figure 5), where *kaiju* and *kraken2* displayed the lowest PRE values, while the remaining profilers showed PREs closer to zero. However, the count distribution in Supplementary Figure S10A spans several orders of magnitude, indicating substantial variability.

To better understand genus-level differences, Supplementary Figure S10B presents the ratio of counts between the weighted average and each profiler. This further highlights the two profiler groups: *kaiju* and *kraken2* show count ratios around 10% compared to the weighted average, while *centrifuge, ganon*, and *krakenuniq* display similar count distributions to the weighted average.

To explore similarities between profilers, we performed a Spearman correlation analysis (Supplementary Figure S11A). The previously mentioned grouping is again evident, although it is noteworthy that despite having similar count levels, *kaiju* and *kraken2* exhibit lower correlation compared to the other profilers. While *ganon, krakenuniq*, and *centrifuge* show strong correlations (≥0.8), the weighted average demonstrates the highest correlation with all profilers. Although this is somewhat expected by definition, it underscores an important point: when in doubt about which profiler to use, the weighted average provides a balanced representation without compromising performance metrics.

The count distribution shows that genera with higher counts tend to have lower dispersion, whereas genera with fewer counts exhibit greater variability. This result, while somewhat expected, highlights that genera with higher counts are more likely to be accurately detected, resulting in greater concordance in reported counts across profilers.

#### 3.3.3 Normalization including biological controls removes putative contaminant genera

One of the main reasons for including biological controls is to identify genera present at abundance levels similar to those found in the samples. Assuming that conditions between the samples and controls have remained stable, we can infer that genera with comparable abundance levels in biological controls and samples are likely the result of contamination during sample processing or, potentially, the presence of microorganisms naturally found on the surfaces of the equipment used.

To determine the extent to which detected genera may be considered contaminants, we applied two types of normalization: (A) combined normalization of plasma-derived EV samples and biological controls, and (B) normalization of plasma-derived EV samples and biological controls separately. The distinction between these approaches is useful because the sequencing coverage of the control samples is approximately 25 times lower than that of the biological samples.

**Figure 9:**
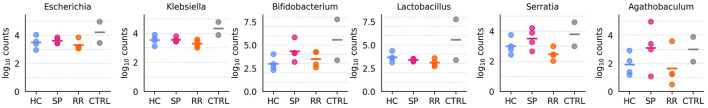
Genera discarded after applying criterion (1), which identified species with similar abundance in both controls and samples without combined normalization. Each plot represents log_10_ transformed counts for each type of sample, with the log_10_ transformed median value depicted in a horizontal bar.

Based on these criteria, we identified three groups of genera: (1) those with similar abundances in both controls and samples without combined normalization, (2) those reclassified as “contaminants” following combined normalization, and (3) those retained after combined normalization, which are assumed to be intrinsic to the biological samples. Out of the 151 initially retained genera, six were eliminated based on criterion (1) (Figure 9), and 61 were discarded based on criterion (2) (Supplementary Figure S12), leaving a total of 84 genera. The genera that are selected or discarded based on these criteria are displayed in Figure 8.

Although some genera may have biological relevance and thus could represent false negatives, our results demonstrate that the combined normalization of samples and controls is both necessary and effective in eliminating unwanted genera. This approach significantly reduces the likelihood of contamination, enhancing the reliability of downstream analyses.

#### 3.3.4 Analysis of differentially abundant genera

Among the remaining 84 genera, we conducted an analysis to identify genera with statistically differential abundance across specific comparisons. Four comparisons were performed: (1) HC vs. SP, (2) HC vs. RR, (3) SP vs. RR, and (4) sex. Given the limited sample size and uncertainty regarding the underlying distribution, we applied a non-parametric test.

**Figure 10:**
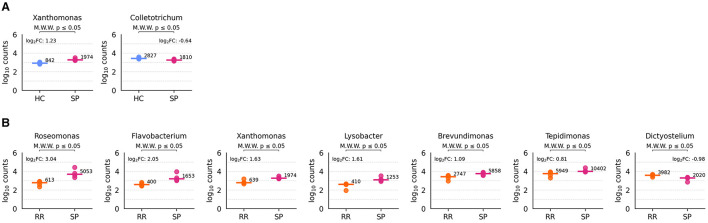
Differentially abundant genera across conditions. The executed comparisons that reported significant genera are HC vs. SP **(A)** and RR vs. SP **(B)**. Each plot represents log_10_ transformed counts for each type of sample, with the log_10_ transformed median value depicted in a horizontal bar.

Significant differences were observed only in the HC vs. SP and RR vs. SP comparisons. Regarding HC vs. SP, *Xanthomonas* showed a significantly higher abundance in SP samples (two-sided Mann-Whitney U test, *U* = 0, *p* = 0.029), while *Colletotrichum* was significantly more abundant in HC samples (*U* = 16, *p* = 0.029) (Figure 10A). In the comparison between RR and SP samples, six genera were more abundant in SP: *Roseomonas, Flavobacterium, Xanthomonas, Lysobacter, Brevundimonas*, and *Tepidimonas* (*U* = 0, *p* = 0.029 for all comparisons). Conversely, *Dictyostelium* was more abundant in RR samples (*U* = 16, *p* = 0.029) (Figure 10B). Of note, none of these *p*-values remained significant after applying the Benjamini-Hochberg correction for multiple testing.

Despite the lack of significance following correction, we also explored genera with *p*-values below 0.15 with high log_2_ fold changes, as these may still provide biologically meaningful insights. These genera are summarized in Supplementary Figure S13. Using this criterion, we identified six genera for HC vs. SP, four for HC vs. RR, 18 for RR vs. SP, and two for sex-related comparisons.

## 4 Discussion

The microbiota has emerged as a critical factor influencing human health, requiring us to expand our focus beyond traditional human-to-human interactions to include the complex dynamics of human-bacteria and bacteria-bacteria interactions (Sender et al., 2016). Additionally, fungi also play an essential role in this equation, as highlighted by their reported involvement in multiple sclerosis (MS) (Mangalam, 2021). Understanding these interactions is crucial to discovering their potential contributions to disease mechanisms.

One powerful tool for exploring these relationships is next-generation sequencing (NGS). However, NGS studies are often plagued by high false-positive rates, particularly when robust methodological controls are not implemented. For example, a recent reanalysis of microbial DNA studies across 33 cancer types demonstrated that lenient thresholds and inadequate host genome mapping led to the detection of implausible bacteria, including extremophiles (Gihawi et al., 2023).

Based on this situation, and with the need to provide a more robust framework to perform taxa profiling, the currently presented workflow aims to reduce some of these biases. The six key aspects derived from this workflow, and which have improved the quality of the analysis, are discussed below.

First, our findings partially align with the observations reported by Gihawi et al. (2023), showing an improvement in detection quality, although not as pronounced as expected or as reported in their study. While false-positive rates were lower than anticipated, many species and their associated counts were correctly assigned without an excessive number of false positives, even in the absence of prior host mapping. It is important to note that the host genome is integrated into the database used by the profilers, which we consider a minimum requirement to reduce the risk of assigning human reads to other organisms. Nonetheless, host mapping remains a valuable step in enhancing the overall accuracy of species detection.

Interestingly, a notable percentage of reads remain unmapped even after host genome mapping, ranging between 4–7% on average and up to 12% in *in silico* datasets. This percentage is also highly dependent on the profiler. This unexplained phenomenon may be due to several reasons including (1) random genome sampling or genomic regions less prone to detection—e.g., mitochondrial genome, centromeric or telomerig regions—which could complicate the identification of less abundant genera, (2) cross-contamination during sample handling or computational artifacts, such as reads aligning to orthologous regions of the human genome, (3) mapping to other genomes not included in the database such as other microorganisms or domestic animals. Further investigation is required to understand this issue and its implications for microbiome studies.

Second, a key aspect of our approach was the creation of a unified database to facilitate a common species/genera detection across different profilers. This step addresses a significant limitation noted by Miceli et al. (2024), who observed that the variability in sample pre-processing and database use impacts the comparability of results in extracellular vesicle (EV) studies. By standardizing the database, we aim to enhance reliability and reduce bias in such detection.

Third, the analysis was designed to minimize the number of parameters and, when included, ensure they are as data-driven as possible adjusted based on the intrinsic properties of the data rather than manual input. The analysis primarily relies on two parameters: the profiling *mode* and the filtering parameter *S*. Notably, we observed greater robustness than expected, particularly for *mode*. Although extreme *mode* values—especially lower ones—can bias read detection by reducing the number of assigned reads, the additional species or genera detected at higher *mode* values appear to be effectively countered by the filtering applied through the *S* parameter. Furthermore, *S* values between 2 and 7 consistently produced stable results in both *in silico* and biological samples. Therefore although parameter tuning may be dataset-dependent, the choice of *S* values between 2 and 7, and *mode* values between 5 to 7, is likely to provide consistent and accurate results.

The key assumption of our current workflow is that genera with fewer assigned counts are less likely to be truly present in the sample and are therefore candidates for removal. This assumption is tied to the compositional nature of microbiome data, where the presence of a few highly abundant taxa alongside many low-abundance taxa amplifies compositional bias (Yang and Chen, 2022). Although there is no consensus on the optimal methods for filtering low-abundance operational taxonomic units (OTUs)—groups of closely related individuals multiple studies have shown that such filtering improves the reliability of OTU detection. Specifically, removing very low-abundance OTUs significantly affects alpha-diversity metrics sensitive to rare genera (e.g., observed OTUs, Chao1) but has minimal impact on the relative abundances of dominant phyla and families or on alpha-diversity metrics that consider both richness and evenness (e.g., Shannon, Inverse Simpson) (Nikodemova et al., 2023). Therefore, incorporating a filtering step to exclude low-abundance genera is recommended.

Fourth, our study also demonstrates that integrating multiple taxa profilers improves detection outcomes. While both in silico and biological datasets show that *centrifuge, ganon*, and *krakenuniq* outperform *kaiju* and *kraken2* in terms of the number of detected reads, combining profilers reduces noise in species counts and enhances detection robustness, particularly in complex datasets. This approach takes advantage of the strengths and variability of different profilers to mitigate individual biases–an often overlooked strategy in routine practice, where a single profiler or the one with the best metrics is typically chosen.

Fifth, and one of the most important aspects of this workflow, is that our results emphasize the critical role of biological controls in reducing false positives. This finding aligns with previous studies, such as Olomu et al. (2020), which highlighted the importance of including controls for reliable EV studies.

Among the most prominent discarded genera in the analysis of joint normalization of biological controls an samples are *Staphylococcus, Cutibacterium*, and *Dermococcus*, some of which have been associated with human skin as commensals (Dessinioti and Katsambas, 2024; Martinson et al., 2019) and are therefore likely contaminants in the samples. Similarly, *Malassezia* is frequently found on scalp skin and is associated with dandruff (Saunte et al., 2020). Ubiquitous genera such as *Escherichia, Serratia*, and *Enterobacter*, although classically considered part of the intestinal microbiota, are also widespread (Fusco et al., 2018) and can be found in saliva, potentially contributing to contamination (Limeres Posse et al., 2017). Finally, some genera are associated with the so-called “kitome", referring to genera inherent to sequencing or library preparation kits (Olomu et al., 2020). Among these, genera such as *Acinetobacter, Lactobacillus*, and *Clostridium* were identified as discarded in this study based on the previous criteria.

However, challenges remain when distinguishing between genera that are both contaminants and naturally present in the study environment. For instance, *Malassezia*, a skin-associated fungus (Saunte et al., 2020), has also been implicated in dysbiosis associated with MS (Mangalam, 2021). Such cases complicate the interpretation of results, as it is difficult to discern whether detected reads originate from the study samples or external contamination. This limitation highlights the need for cautious interpretation of results and the careful selection of thresholds to avoid excessive filtering of potentially relevant genera.

Lastly, despite these limitations, our analysis identified promising candidates for further investigation within both MS and control groups. This is particularly relevant given documented shifts in the stool microbiome of MS patients (Thirion et al., 2023; Zhou et al., 2022). Interestingly, only a small number of genera exhibited differential abundance between groups specially between RR and SP samples (7 genera). Despite not being significant, *Blautia* showed a relatively increased abundance in MS samples compared to healthy controls (log2FC of 6.2 and 4.1, *p*-values of 0.057 and 0.11 for SP vs. HC and RR vs. HC), consistent with findings by Ghezzi et al. (2021), while Cox et al. (2021) reported a decrease in MS samples. Similarly, a non-significant increase in *Sutterella* abundance was observed in SP samples, contrasting with the decrease reported by Ghezzi et al. (2021). These discrepancies highlight the challenges in comparing results across studies due to differences in sample type (stool vs. EVs) and analysis approach (16S rRNA vs. RNA-seq). Finally, considering SP vs. HC comparison, we observed a significant abundance of *Colletotrichum* in HC samples. Colletotrichamide C, a metabolite from specific species of the *Colletotrichum* genera, exhibited strong neuroprotective activity against glutamate neurotoxicity implicated in chronic neurodegenerative diseases, such as MS (Bang et al., 2019).

This study presents a powerful new workflow designed to reorient the analysis of RNAseq data from plasma EVs, enabling the efficient identification of relevant bacterial transcripts. Beyond plasma EVs, this approach can be applied to other total RNA samples, leading to more accurate microbial genus identification and a reduction in false positive results. While bacterial transcripts were successfully identified, further research will be crucial to determine if they are delivered via putative bEVs in blood and to explore the specific genes and functions they encode.

## 5 Study limitations

The study may suffer from the following limitations. Although the idea of the project is a proof of concept for the analysis tools, the results of the differentially abundant genera must be taken with caution due to the size of the groups. Additionally, the variation within the biological controls has to be considered. Therefore, more biological samples and controls should be included. Secondly, and as has been reported by Miceli et al. (2024), the amount of genetic material in EVs is scarce, and thus discovery and profiling of non-human species may be hindered by this fact. Additionally, these results, but not the analysis flow proposed, are dependent on the methodology used to isolate the EVs, a common issue for all EV studies. Lastly, it should be considered that part of the retrieved bacterial reads could not be directly associated with the presence of bEVs, but rather from other forms of genetic material including RNA derived from blood-circulating bacteria.

## Data Availability

The datasets presented in this study can be found in online repositories. The names of the repository/repositories and accession number(s) can be found below: https://www.ncbi.nlm.nih.gov/geo/, GSE255317, https://doi.org/10.5281/zenodo.14887264, doi: 10.5281/zenodo.14887264.
