# Supplementary material for: A proposed workflow to robustly analyze bacterial transcripts in RNAseq data from extracellular vesicles
Source: Front Microbiol. 2025 Mar 20;16:1486661. doi: 10.3389/fmicb.2025.1486661 (PMC11981554; doi:10.3389/fmicb.2025.1486661)
Supplement: Supplementary file 1 [file Data_Sheet_1.pdf]

A proposed workflow to robustly analyze bacterial transcripts in RNAseq data from Extracellular Vesicles - Supplementary Information

Alex M. Ascensión, Miriam Gorostidi-Aicua, Ane Otaegui-Chivite, Ainhoa Alberro, Rocio del Carmen Bravo-Miana, Tamara Castillo-Trivino, Laura Moles and David Otaegui

1 Supplementary Tables

Table S1: Main clinical and demographical characteristics of individuals enrolled in the study classified by disease status.

Disease status	Sex	Age	EDSS	Evol. time	AOO
RR (n=10)	Male (n=5)	39.7 (±13.7)	2 (0-3.5)	16.1 (±9.8)	23.6 (±9.1)
	Female (n=5)	42.0 (±19.8)	0 (0-2)	13.2 (±12.2)	28.8 (±9.9)
SP (n=10)	Male (n=5)	54.8 (±6.2)	6 (6-8.5)	20.7 (±8.7)	34.1 (±9.5)
	Female (n=5)	48.7 (±6.7)	7 (4-8)	22.5 (±5.0)	26.2 (±6.6)
HC (n=8)	Male (n=4)	56.1 (±11.9)	-	-	-
	Female (n=4)	53.6 (±11.8)	-	-	-

Abbreviations: RR, relapsing-remitting multiple sclerosis; SP. Secondary-progressive multiple sclerosis; HC, healthy control; EDSS, Expanded Disability Status Scale; Evol. Time: Evolution time; AOO, Age of Onset. Age, evolution time and AOO data are presented as “average (standard deviation)”, EDSS data are shown as “median (range)”.

Table S2: Patient description of each pool.

**Pool name**	**Patient**	**Sample collection**	**Disease Status**	**Sex**
Pool 1	EVRR1	2/18/2010	RR	M
	EVRR2	2/25/2012	RR	M
	EVRR3	4/23/2009	RR	M
Pool 2	EVRR4	2/25/2010	RR	M
	EVRR5	6/19/2013	RR	M
Pool 3	EVRR6	12/2/2010	RR	F
	EVRR7	5/26/2009	RR	F
	EVRR8	7/9/2009	RR	F
Pool 4	EVRR9	7/9/2009	RR	F
	EVRR10	9/16/2010	RR	F
Pool 5	EVSP1	3/5/2009	SP	M
	EVSP2	5/28/2009	SP	M
	EVSP3	3/28/2011	SP	M
Pool 6	EVSP4	12/23/2010	SP	M
	EVSP5	4/28/2009	SP	M
Pool 7	EVSP6	2/17/2011	SP	F
	EVSP7	9/12/2000	SP	F
	EVSP8	9/28/2010	SP	F
Pool 8	EVSP9	1/13/2011	SP	F
	EVSP10	10/7/2010	SP	F
Pool 9	EVHC1	12/2/2004	HC	M
	EVHC2	9/22/2009	HC	M
Pool 10	EVHC3	9/24/2009	HC	M
	EVHC4	9/24/2009	HC	M
Pool 11	EVHC5	12/16/2009	HC	F
	EVHC6	2/26/2009	HC	F
Pool 12	EVHC7	7/4/2011	HC	F
	EVHC8	7/15/2011	HC	F

Table S3: Summary table of species used to generate the artificial reads. Several genera contain more than one species, which are marked with an asterisk.

Kingdom	Species	NCBI Tax ID	N reads	Rel. abundance (%)
Animal	Homo sapiens	9606	40000000	80
Bacteria	Cutibacterium acnes	1747	312500	0.625
Bacteria	Lactobacillus acidophilus	1579	312500	0.625
Bacteria	Bifidobacterium bifidum	1681	312500	0.625
Bacteria	Akkermansia muciniphila	239935	312500	0.625
Bacteria	Blautia coccoides (*)	1532	312500	0.625
Bacteria	Blautia luti (*)	89014	312500	0.625
Bacteria	Bacteroides ovatus (*)	28116	312500	0.625
Bacteria	Bacteroides intestinalis (*)	329854	312500	0.625
Bacteria	Bacteroides fragilis (*)	817	312500	0.625
Bacteria	Escherichia coli	511145	312500	0.625
Bacteria	Dietzia lutea	546160	250000	0.5
Bacteria	Ruthenibacterium lactatiformans	1550024	187500	0.375
Bacteria	Faecalibacterium prausnitzii	853	187500	0.375
Bacteria	Parabacteroides distasonis (*)	823	187500	0.375
Bacteria	Parabacteroides merdae (*)	46503	187500	0.375
Bacteria	Fusicatenibacter saccharivorans	1150298	187500	0.375
Bacteria	Erysipelatoclostridium ramosum	1547	125000	0.25
Bacteria	Streptococcus salivarius	1304	125000	0.25
Bacteria	Hungatella hathewayi	154046	62500	0.125
Bacteria	Eisenbergiella porci	2652274	62500	0.125
Bacteria	Butyricimonas faecalis	2093856	62500	0.125
Bacteria	Alistipes indistinctus (*)	626932	62500	0.125
Bacteria	Alistipes finegoldii (*)	214856	62500	0.125
Bacteria	Eubacterium callanderi	53442	62500	0.125
Bacteria	Acidaminococcus intestini	187327	62500	0.125
Fungi	Aspergillus chevalieri (*)	182096	200000	0.4
Fungi	Aspergillus flavus (*)	5059	200000	0.4
Fungi	Saccharomyces cerevisiae (*)	4932	200000	0.4
Fungi	Saccharomyces kudriavzevii (*)	114524	200000	0.4
Fungi	Saccharomyces mikatae (*)	114525	200000	0.4
Fungi	Candida albicans (*)	5476	200000	0.4
Fungi	Candida dubliniensis (*)	42374	200000	0.4
Fungi	Candida orthopsilosis (*)	273371	200000	0.4
Fungi	Malassezia restricta	76775	150000	0.3
Fungi	Alternaria dauci	48095	150000	0.3
Fungi	Kazachstania africana	432096	100000	0.2
Fungi	Penicillium digitatum	36651	100000	0.2
Fungi	Pichia kudriavzevii	4909	50000	0.1
Fungi	Trichoderma asperellum	101201	50000	0.1
Fungi	Akanthomyces muscarius	2231603	50000	0.1
Fungi	Fusarium falciforme	195108	50000	0.1
Fungi	Eremothecium sinecaudum	45286	50000	0.1
Fungi	Cryptococcus decagattii	1859122	50000	0.1
Fungi	Kwoniella shandongensis	1734106	50000	0.1
Fungi	Puccinia triticina	208348	50000	0.1
Virus	Tobacco mosaic virus	12242	250000	0.5
Virus	Rotavirus A (*)	28875	250000	0.5
Virus	Rotavirus B (*)	28876	250000	0.5
Virus	Rotavirus C (*)	36427	250000	0.5
Virus	Bacteriophage P2	10679	200000	0.4
Virus	Escherichia phage T4	10665	200000	0.4
Virus	Human immunodeficiency virus 1	11676	200000	0.4
Virus	Human adenovirus 7	108098	150000	0.3
Virus	Hepatitis C virus	3052230	150000	0.3
Virus	Bovine alphaherpesvirus 2	3050244	150000	0.3
Virus	Human herpesvirus 4 type	3050299	150000	0.3
Virus	Mimivirus terra2	1128151	100000	0.2
Virus	Dengue virus	3052464	100000	0.2
Virus	Norovirus GI	11983	50000	0.1
Virus	Zaire ebolavirus	3052462	50000	0.1

Table S4: Parameter values for each profiler and each profiling mode.

Profiler	Argument	Mode 1	Mode 2	Mode 3	Mode 4	Mode 5	Mode 6	Mode 7	Mode 8	Mode 9
Kraken2	confidence	0.9	0.85	0.8	0.75	0.7	0.65	0.6	0.55	0.5
Krakenuniq	hll-precision	18	17	16	15	14	13	12	11	10
Centrifuge	min-hitlen	51	47	43	39	35	31	27	23	19
Ganon	rel-cutoff	0.9	0.9	0.8	0.8	0.7	0.7	0.6	0.6	0.5
Ganon	rel-filter	0.4	0.35	0.3	0.25	0.2	0.15	0.1	0.05	0
Kaiju	E	0.001	0.001	0.01	0.01	0.01	0.05	0.05	0.1	0.1
Kaiju	m	43	39	35	31	27	23	19	15	11
Kaiju	e	2	2	3	3	4	4	4	5	5
KMCP | search + profile	min-query-cov	0.75	0.7	0.65	0.6	0.55	0.5	0.45	0.4	0.35
KMCP | search + profile	max-fpr	0.001	0.001	0.01	0.01	0.01	0.05	0.05	0.1	0.1
KMCP | search	min-query-len	43	39	35	31	27	23	19	15	11
KMCP | profile	mode	0	0	1	2	3	3	4	4	5

Table S5: Sample information and percentages of reads used in the next step of the processing pipeline. For each cell within the profiler, the two values represent the percentage maped using modes 3 and 7 respectively. For instance, POOL1 sample originally contained 56.55 million of reads (100%). Of that amount, 6.67% (3.77M) were retained after the first map (the rest were mapped to human); and 5.81% (3.28M) were retained after the second map. Lastly of these reads, 0.2% were mapped to human by *kraken2* usign mode3, 14.39% were mapped to non-human genera, and 5.58% remained unmapped.
Column labels: CEN (*centrifuge*), GAN (*ganon*), KAI (*kaiju*), KR2 (*kraken2*), KRU (*krakenuniq)*.

				**MAPPED TO HUMAN (%)**				
	**# reads**	**1st map (%)**	**2nd map (%)**	**CEN**	**GAN**	**KAI**	**KR2**	**KRU**
**ARTIFICIAL**	49996111	24.945	23.554	2.12, 2.14	2.35, 2.46	0.14, 0.34	1.18, 1.63	2.19, 2.19
**ACIDOLA**	2463392	96.671	96.660	0.0008, 0.008	0.0219, 0.0816	0.0, 0.0	0.0, 0.0	0.103, 0.103
**BLACTIS**	2109117	99.207	99.206	0.0001, 0.0009	0.0013, 0.0059	0.0, 0.0	0.0, 0.0	0.0135, 0.0135
**POOL1**	56548742	6.669	5.795	3.03, 4.44	1.49, 2.59	1.1, 1.78	0.2, 1.06	4.71, 4.71
**POOL2**	42205676	11.649	10.577	3.55, 4.71	1.56, 2.87	1.19, 1.93	0.24, 1.11	4.99, 4.99
**POOL3**	48792251	9.773	6.805	1.93, 3.29	1.34, 2.2	0.58, 1.05	0.12, 0.76	3.7, 3.7
**POOL4**	47145951	9.114	6.114	2.52, 3.87	1.76, 2.84	0.68, 1.29	0.15, 1.12	4.32, 4.32
**POOL5**	44694021	8.213	6.213	1.71, 2.63	1.12, 1.88	0.47, 0.92	0.1, 0.66	2.91, 2.91
**POOL6**	55640398	43.863	42.758	1.02, 1.41	0.63, 1.05	0.27, 0.55	0.07, 0.39	1.55, 1.55
**POOL7**	55697936	9.380	7.274	1.62, 2.52	1.23, 1.93	0.41, 0.76	0.11, 0.65	2.84, 2.84
**POOL8**	47826994	29.821	28.570	1.67, 2.73	1.02, 1.68	0.49, 0.92	0.11, 0.69	2.96, 2.96
**POOL9**	42852541	10.349	8.684	1.79, 2.58	1.26, 1.98	0.55, 0.95	0.16, 0.73	2.82, 2.82
**POOL10**	55067711	8.995	5.425	1.85, 2.9	1.32, 2.17	0.57, 0.98	0.13, 0.74	3.31, 3.31
**POOL11**	41656703	7.726	5.224	1.61, 2.95	1.07, 1.74	0.45, 0.82	0.12, 0.63	3.24, 3.24
**POOL12**	40367876	11.125	9.099	1.95, 3.4	1.62, 2.56	0.48, 0.88	0.13, 0.85	3.87, 3.87

	**MAPPED TO NON-HUMAN (%)**					**UNMAPPED (%)**				
	**CEN**	**GAN**	**KAI**	**KR2**	**KRU**	**CEN**	**GAN**	**KAI**	**KR2**	**KRU**
**ARTIFICIAL**	16.0, 16.3	18.08, 19.48	10.23, 11.21	8.66, 11.3	16.13, 16.13	5.44, 5.11	3.12, 1.61	13.18, 12.01	13.72, 10.62	5.24, 5.24
**ACIDOLA**	88.31, 89.3	90.87, 94.18	16.66, 20.13	13.64, 21.83	88.45, 88.45	8.35, 7.35	5.77, 2.4	80.0, 76.53	83.02, 74.83	8.11, 8.11
**BLACTIS**	74.29, 75.22	92.87, 97.34	0.4, 0.48	14.39, 32.72	74.52, 74.52	24.92, 23.98	6.33, 1.86	98.81, 98.72	84.82, 66.49	24.67, 24.67
**POOL1**	0.35, 0.42	0.23, 0.34	0.07, 0.17	0.02, 0.03	0.36, 0.36	2.42, 0.94	4.08, 2.86	4.63, 3.85	5.58, 4.71	0.72, 0.72
**POOL2**	1.6, 2.11	1.43, 2.1	0.45, 1.0	0.07, 0.15	2.07, 2.07	5.42, 3.76	7.59, 5.6	8.94, 7.64	10.27, 9.32	3.52, 3.52
**POOL3**	0.87, 1.11	1.08, 1.44	0.14, 0.34	0.04, 0.08	1.04, 1.04	4.01, 2.4	4.39, 3.17	6.08, 5.41	6.64, 5.97	2.06, 2.06
**POOL4**	0.48, 0.59	0.51, 0.66	0.08, 0.2	0.03, 0.06	0.51, 0.51	3.12, 1.66	3.84, 2.61	5.35, 4.62	5.93, 4.94	1.28, 1.28
**POOL5**	0.81, 1.05	1.29, 1.65	0.18, 0.4	0.04, 0.09	1.03, 1.03	3.69, 2.53	3.81, 2.68	5.56, 4.9	6.06, 5.47	2.27, 2.27
**POOL6**	18.88, 20.89	24.69, 31.27	1.79, 3.47	0.87, 1.85	20.36, 20.36	22.85, 20.46	17.44, 10.43	40.69, 38.74	41.82, 40.52	20.85, 20.85
**POOL7**	1.18, 1.53	1.66, 2.2	0.24, 0.56	0.05, 0.11	1.51, 1.51	4.48, 3.22	4.38, 3.15	6.62, 5.95	7.11, 6.52	2.93, 2.93
**POOL8**	10.74, 12.18	15.74, 19.93	0.55, 1.13	0.21, 0.58	11.83, 11.83	16.16, 13.66	11.8, 6.96	27.53, 26.52	28.25, 27.3	13.78, 13.78
**POOL9**	1.61, 2.03	2.53, 3.27	0.32, 0.85	0.07, 0.15	2.13, 2.13	5.29, 4.07	4.9, 3.43	7.81, 6.89	8.46, 7.81	3.74, 3.74
**POOL10**	0.52, 0.67	0.73, 0.95	0.1, 0.22	0.02, 0.05	0.62, 0.62	3.06, 1.86	3.37, 2.31	4.76, 4.22	5.27, 4.63	1.5, 1.5
**POOL11**	0.47, 0.62	0.57, 0.75	0.09, 0.22	0.02, 0.04	0.58, 0.58	3.14, 1.65	3.58, 2.73	4.68, 4.19	5.08, 4.55	1.4, 1.4
**POOL12**	1.38, 1.77	2.08, 2.67	0.26, 0.6	0.07, 0.16	1.74, 1.74	5.76, 3.93	5.41, 3.87	8.36, 7.61	8.9, 8.09	3.48, 3.48

Table S6: Detection of genera from biological samples and controls, depending on the *mode* parameter. The ratios between modes 7 to 3, and 5 to 3; and the percentage of genera from *mode*3 that are detected by *mode*5/*mode*7 are also included.

	**Mode 3**	**Mode 5**	**Mode 7**	**Ratio 5 / 3**	**Ratio 7 / 3**	**% 3 in 5**	**% 3 in 7**
ACIDOLA	563	603	668	1.071	1.187	99.112	98.401
BLACTIS	163	194	253	1.190	1.552	95.706	93.865
HC1	2087	2129	2186	1.020	1.047	99.473	99.281
HC2	1995	2054	2139	1.030	1.072	99.499	99.599
HC3	1956	1996	2088	1.020	1.067	99.335	99.335
HC4	2160	2204	2260	1.020	1.046	99.630	99.259
RR1	1449	1527	1622	1.054	1.119	99.517	99.379
RR2	1808	1871	1955	1.035	1.081	99.226	99.004
RR3	1955	2000	2081	1.023	1.064	99.130	99.028
RR4	1951	1999	2076	1.025	1.064	99.334	99.026
SP1	1919	1982	2038	1.033	1.062	99.635	99.583
SP2	2235	2282	2344	1.021	1.049	99.776	99.732
SP3	1934	1982	2061	1.025	1.066	99.638	99.431
SP4	2314	2354	2408	1.017	1.041	99.827	99.697

2 Supplementary figures

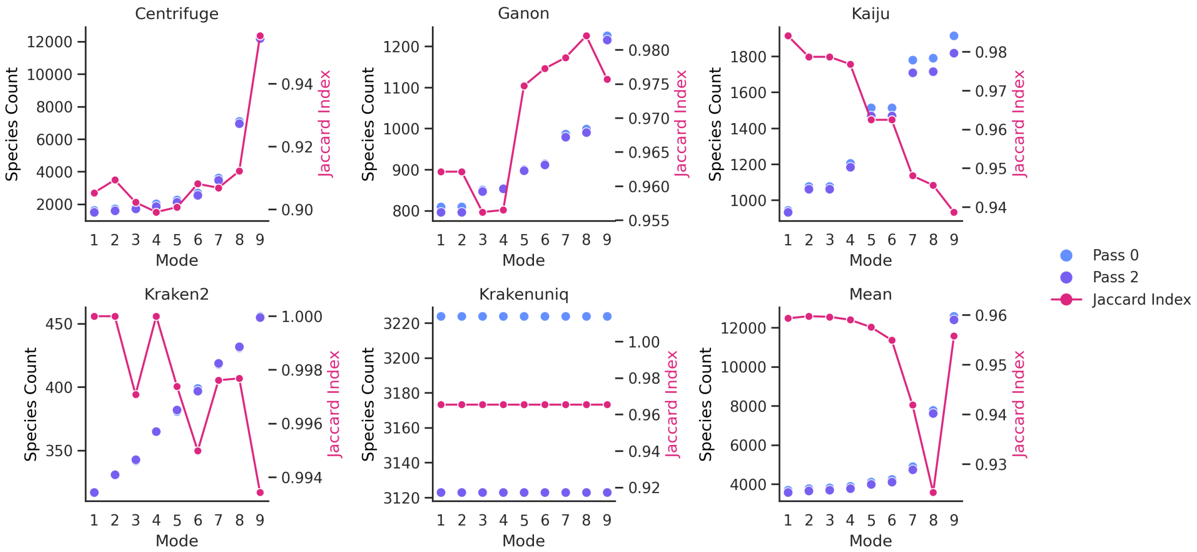

Figure S1: Jaccard index comparison of detected species across profiling modes for the *in silico* dataset. Left y axis indicates the total number of species detected as pass2 and pass0; and right y axis represents the Jaccard index between the number of detected species for each pass.

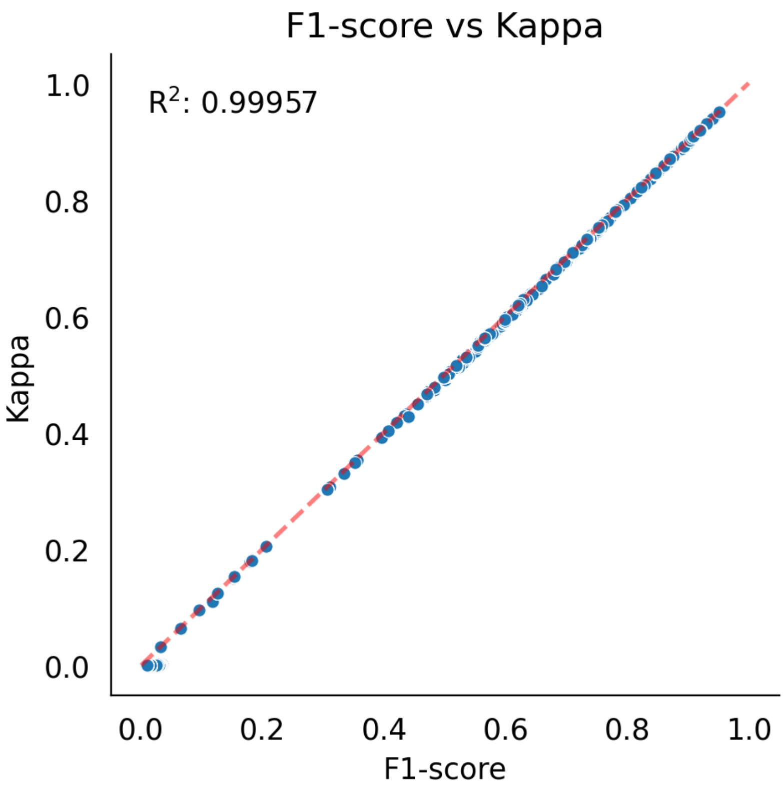

Figure S2: Correlation between the F1-score and the κ value for the evaluated 540 combinations of *mode*, S and profiler.

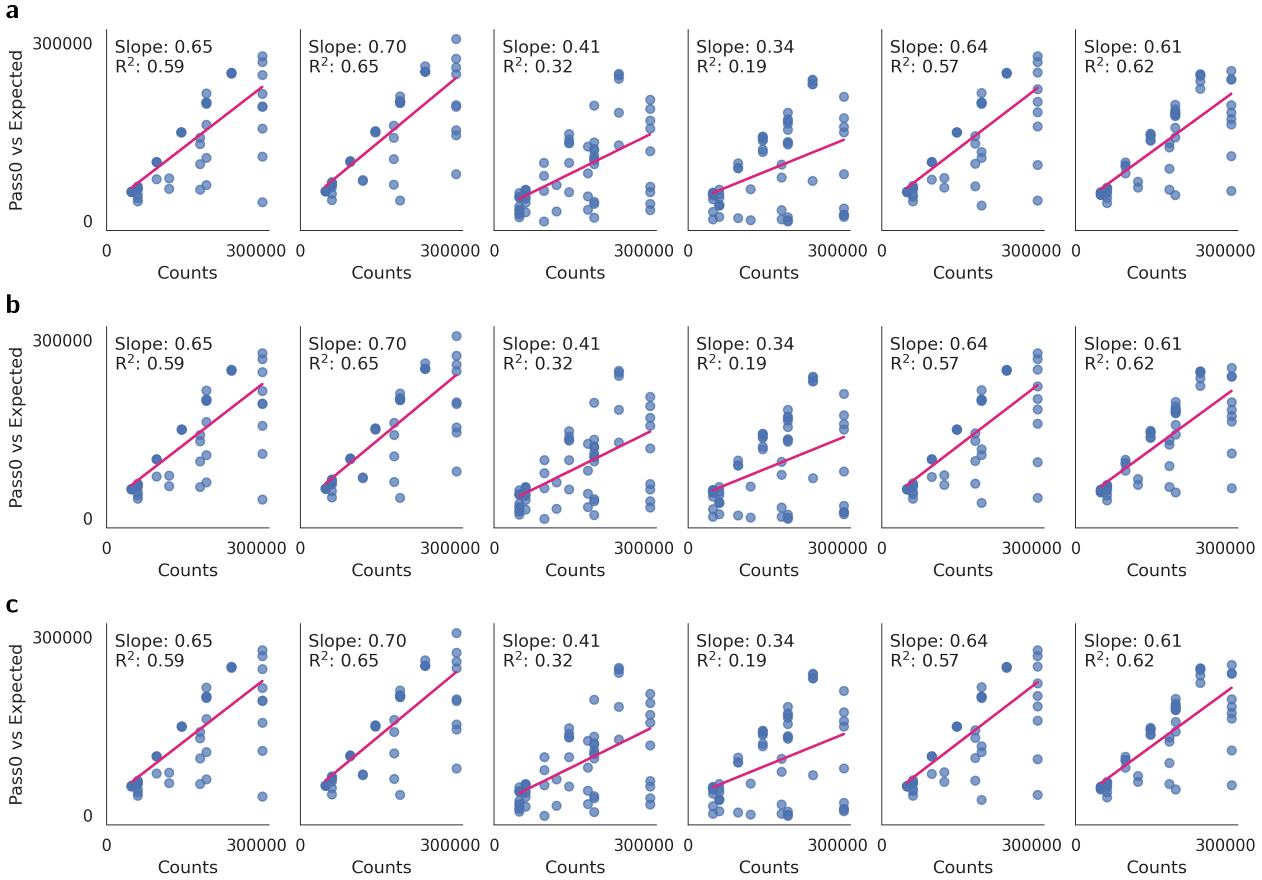

Figure S3: Correlation plots for three scenarios: (a) pass0 vs. pass2 observed counts, (b) pass2 observed counts vs. expected counts and (c) pass0 observed counts vs. expected counts. The figure highlights the y=x dotted line in (a), and the linear regression lines for (b) and (c), with the regression slope and Pearson correlation coefficient.

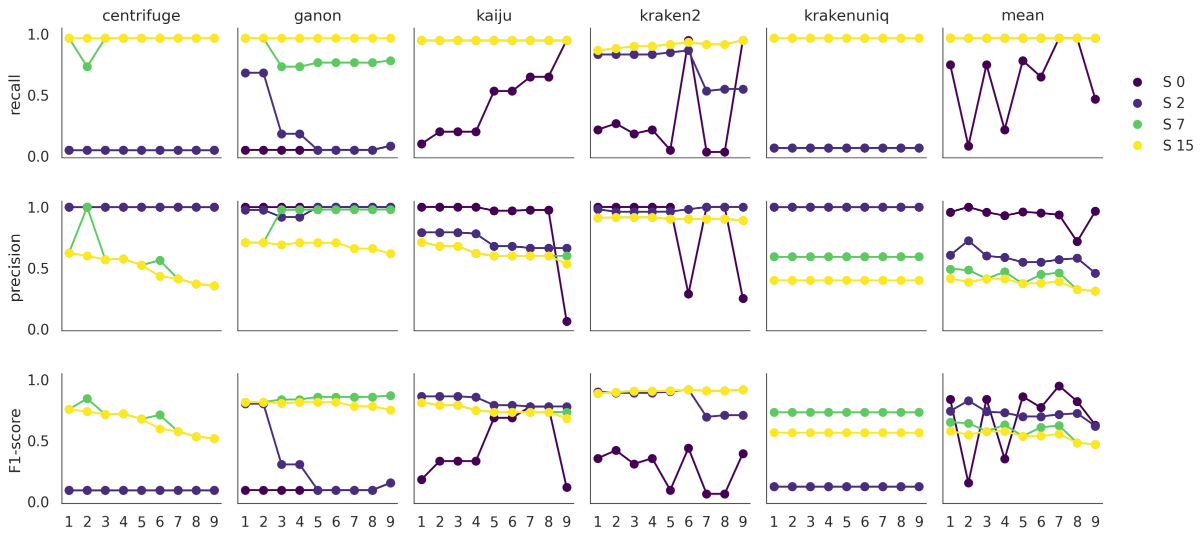

Figure S4: Impact of the *mode* parameter on recall, precision, and F1 score. For each plot, the x-axis represents the *mode* value, each line is a different S value.

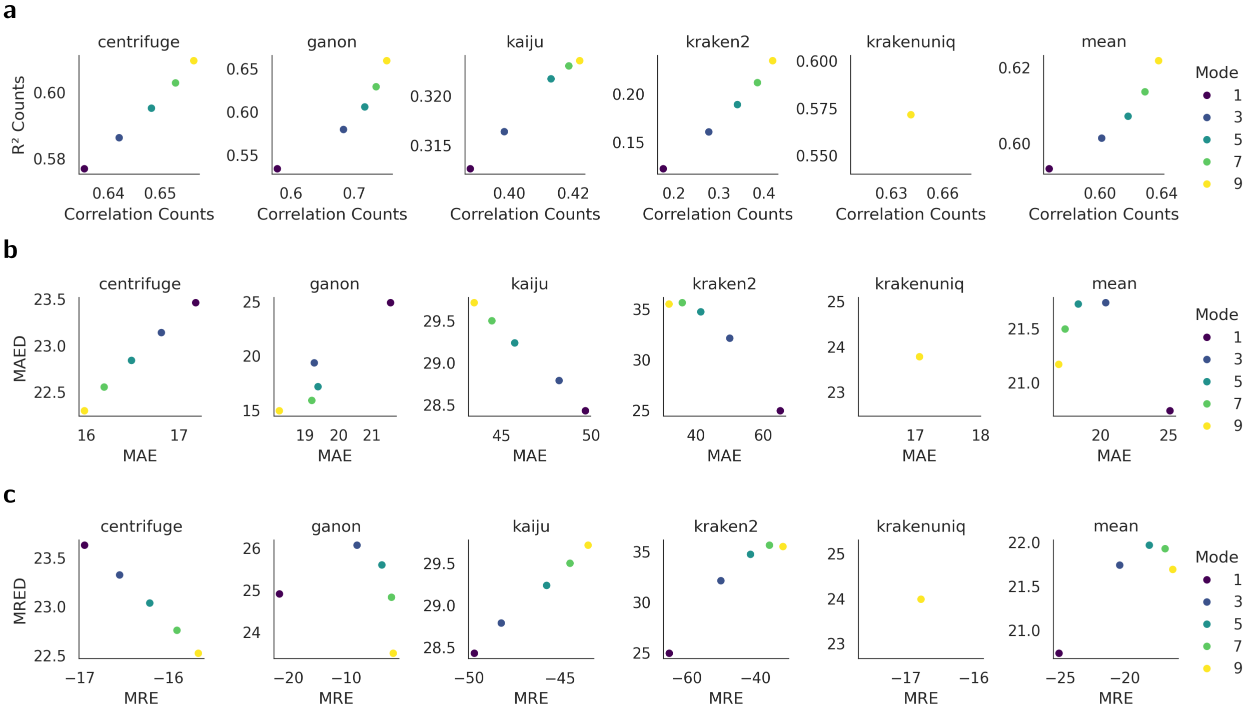

Figure S5: Performance of *mode* values across profilers. (a) slope of the regression for expected versus observed counts vs. Correlation of counts (R^2), (b) mean absolute error (MAE) vs. mean absolute error deviation (MAED), and (c) mean relative error (MRE) vs. mean relative error deviation (MRED).

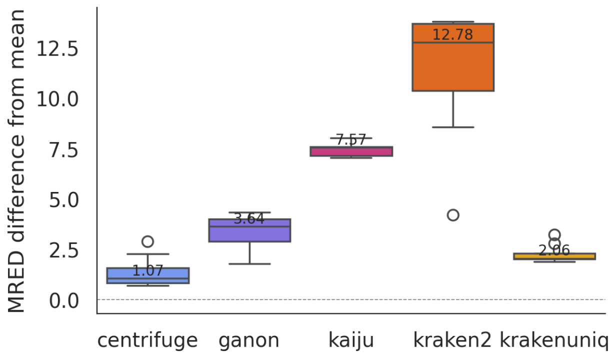

Figure S6: Differences in MRED between weighted average counts and other profilers. Each difference is computed as the difference for the same *mode* value. Median value of differences across *mode* values is present in each box.

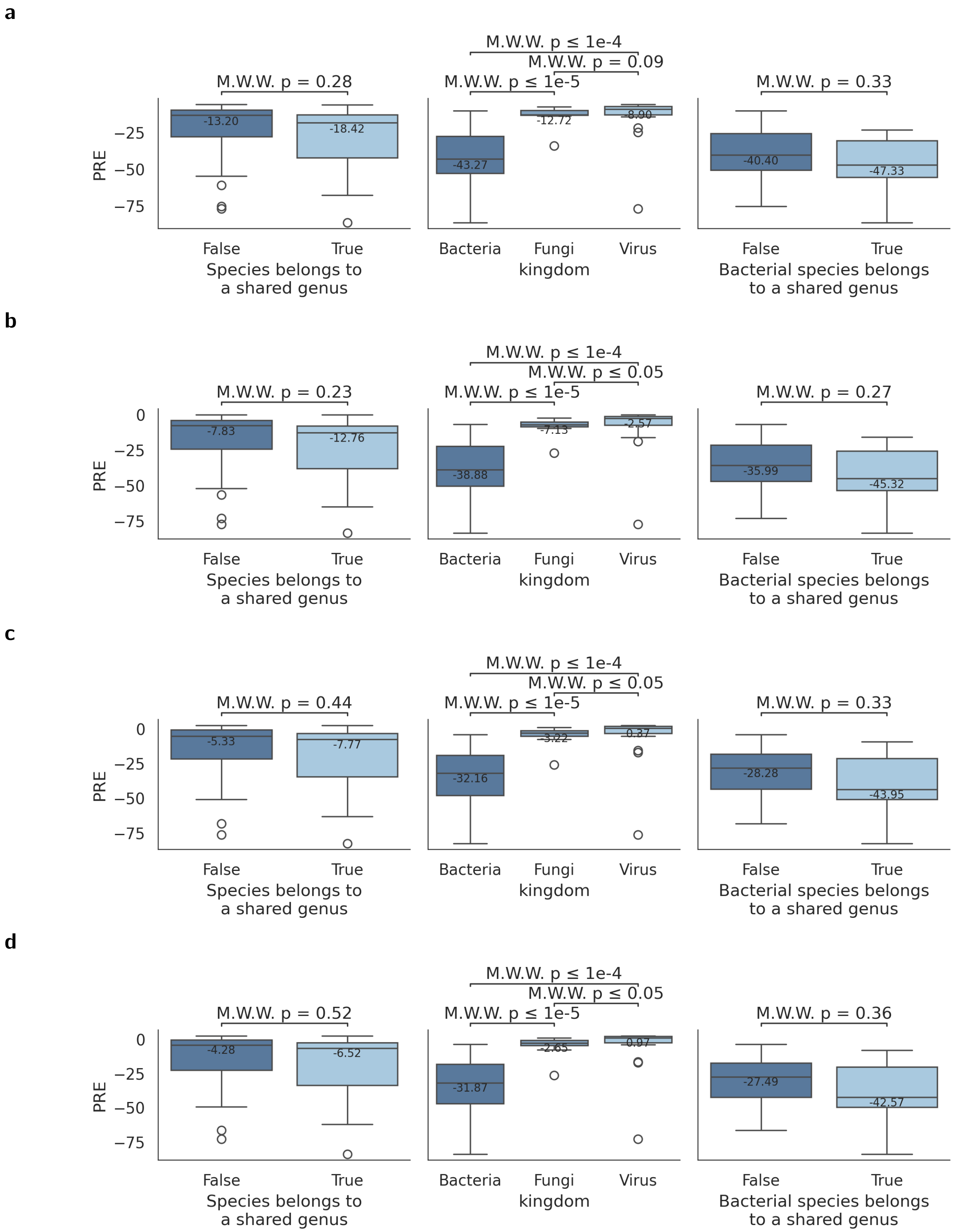

Figure S7: Differences in PRE across species and genera for different *mode* values, using weighted average counts. Boxplots are represented for *mode* 1 (a), 3 (b), 7 (c) and 9 (d). Left boxplot indicates PRE values for species that belong to a shared genus (True) vs. those who do not (False). Right plot shows the same information, but restricted to bacterial species. Mid box plot represents PRE values for *Bacteria*, *Fungi*, and *Virus*.

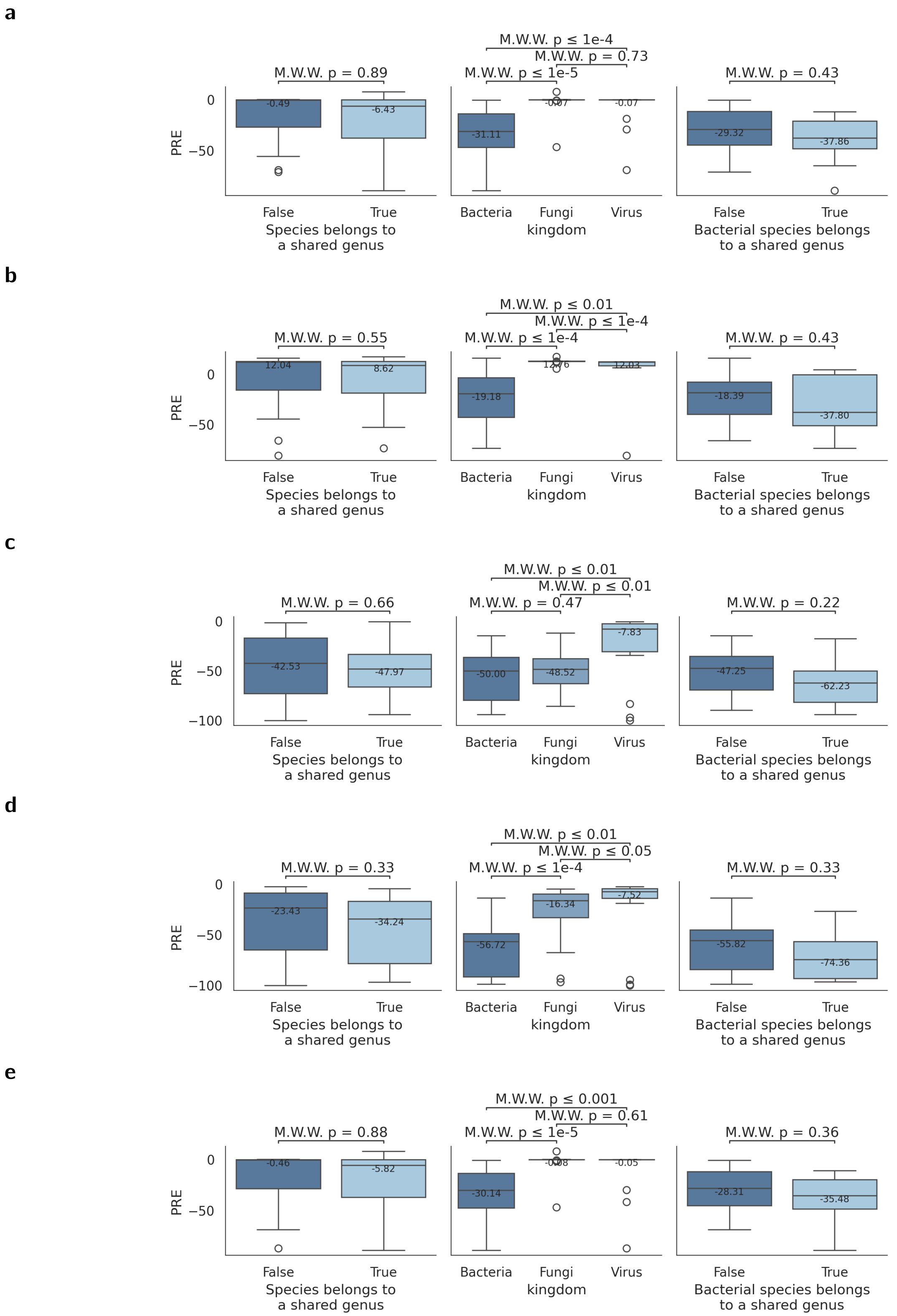

Figure S8: Differences in PRE across species and genera for different profilers, with *mode* 5. Boxplots are represented for *centrifuge* (a), *ganon* (b), *kaiju* (c), *kraken2* (d) and *krakenuniq* (e). Left boxplot indicates PRE values for species that belong to a shared genus (True) vs. those who do not (False). Right plot shows the same information, but restricted to bacterial species. Mid box plot represents PRE values for *Bacteria*, *Fungi*, and *Virus*.

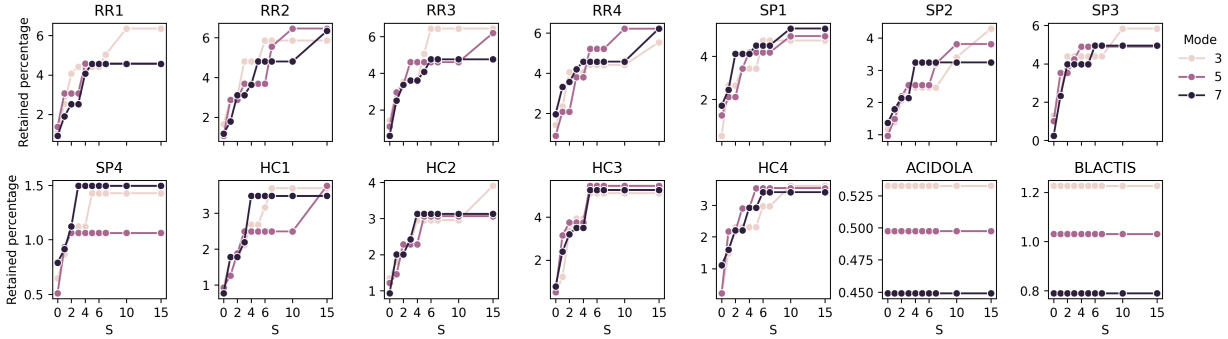

Figure S9: Retention percentage of genera across different S values. Each subplot corresponds to a specific sample, and each line represents a different *mode* value.

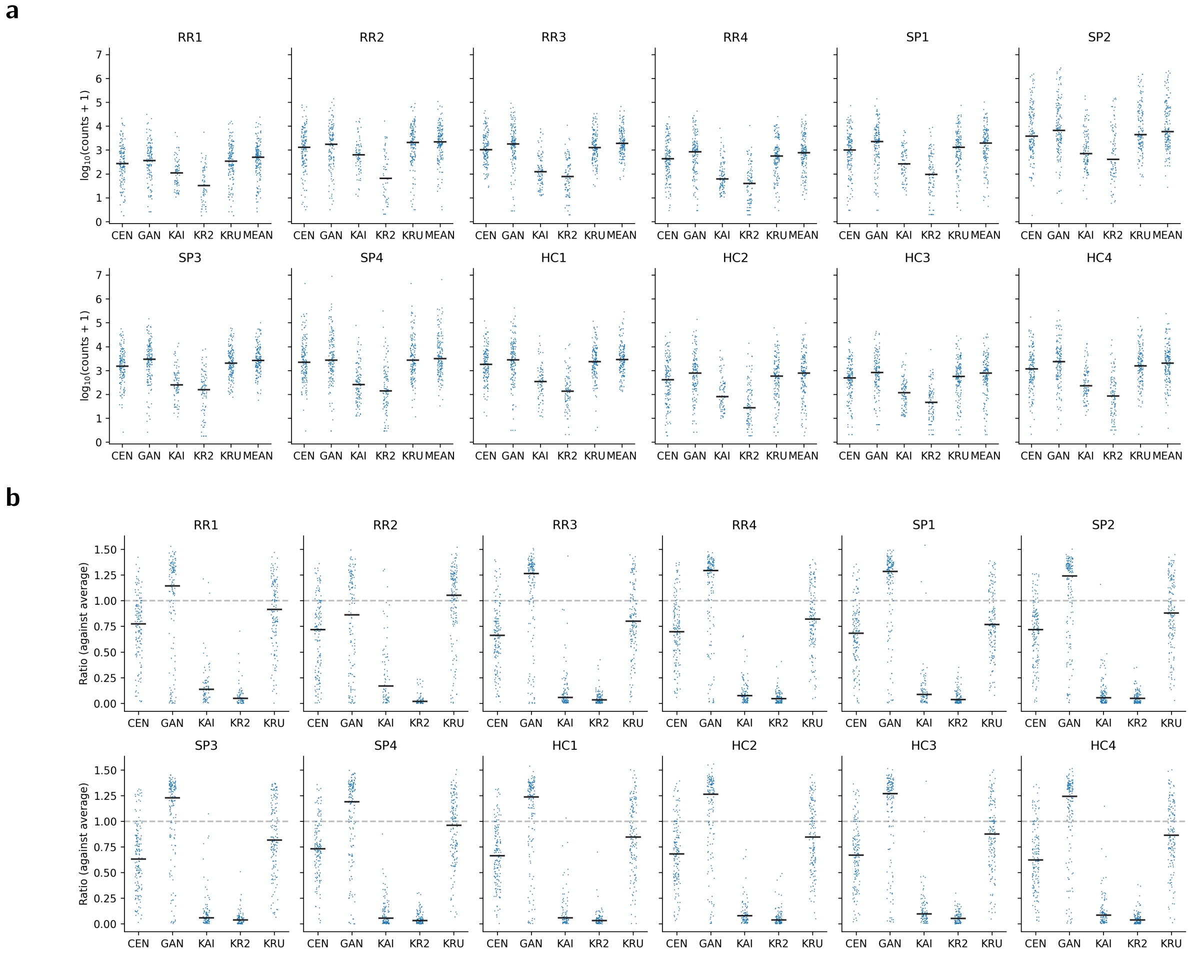

Figure S10: (a) Strip plot of $\log_{10}(\text{counts} + 1)$ of the 151 selected genera from biological samples. Gray bars represent the median value. (b) Strip plot of the ratio of counts–not log(counts)–between each profiler and the weighted average for each genus.

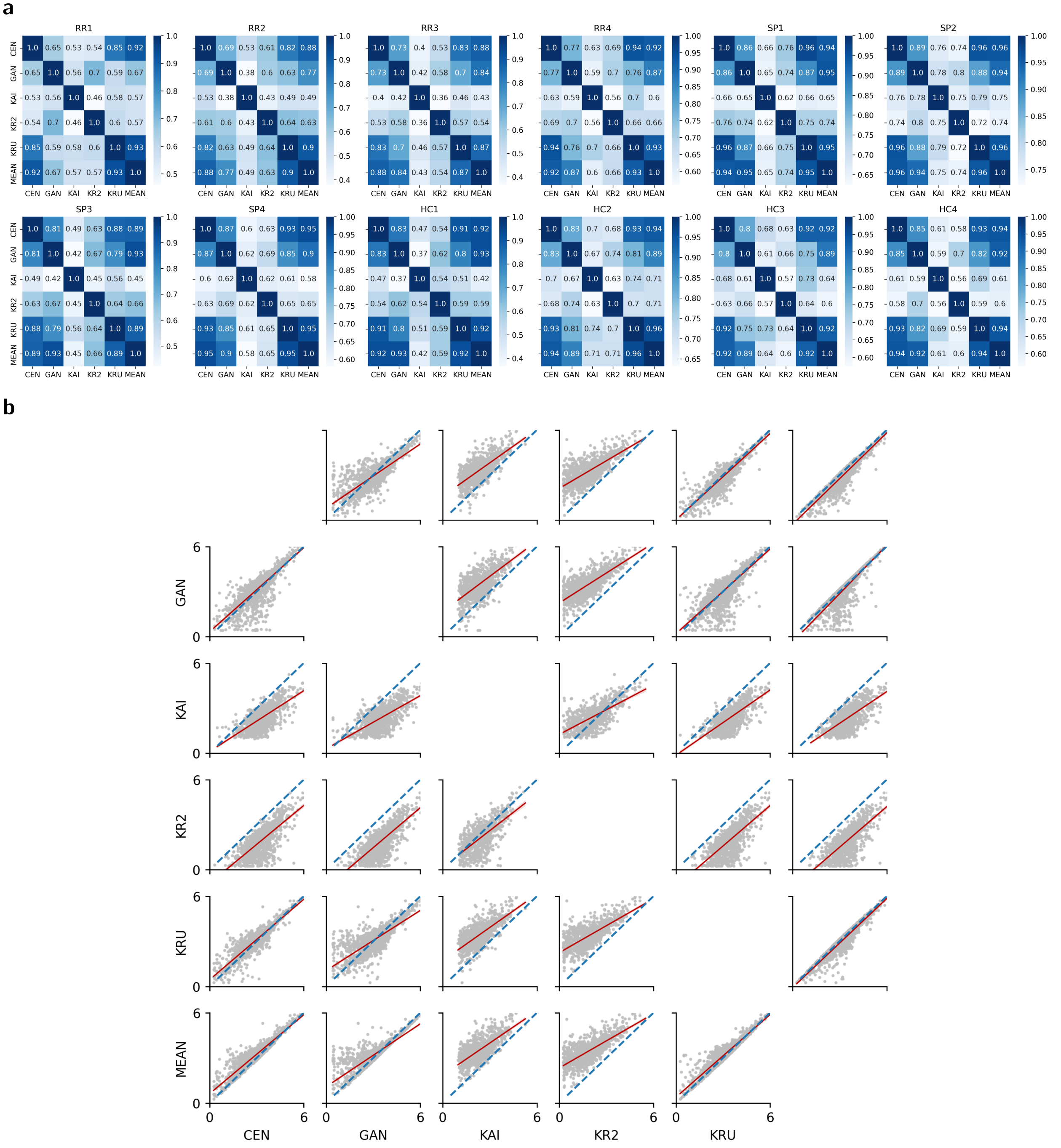

Figure S11: (a) Heatmaps of the Spearman correlation of the number of counts from the 151 selected genera from biological samples. (b) Correlation plot of the $\log_{10}(\text{counts} + 1)$ of the counts from (a), with counts from all samples combined. Dashed blue line represents the identity line y=x, and the red line represents the linear regresion of the data.

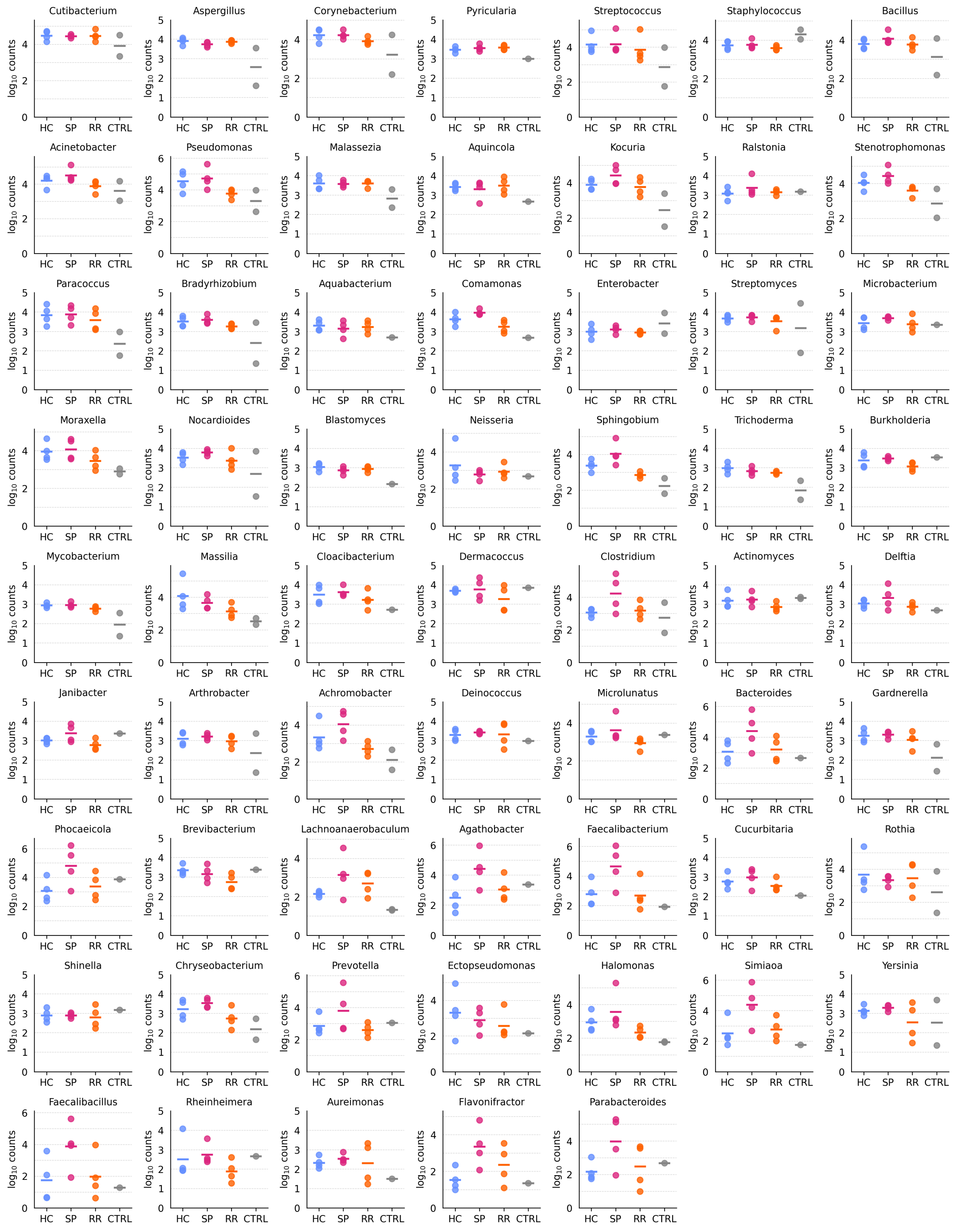

Figure S12: Genera discarded after applying criterion (2), reclassified as “contaminants” following combined normalization. Each plot represents $\log_{10}$ transformed counts for each type of sample, with the $\log_{10}$ transformed median value depicted in a horizontal bar.

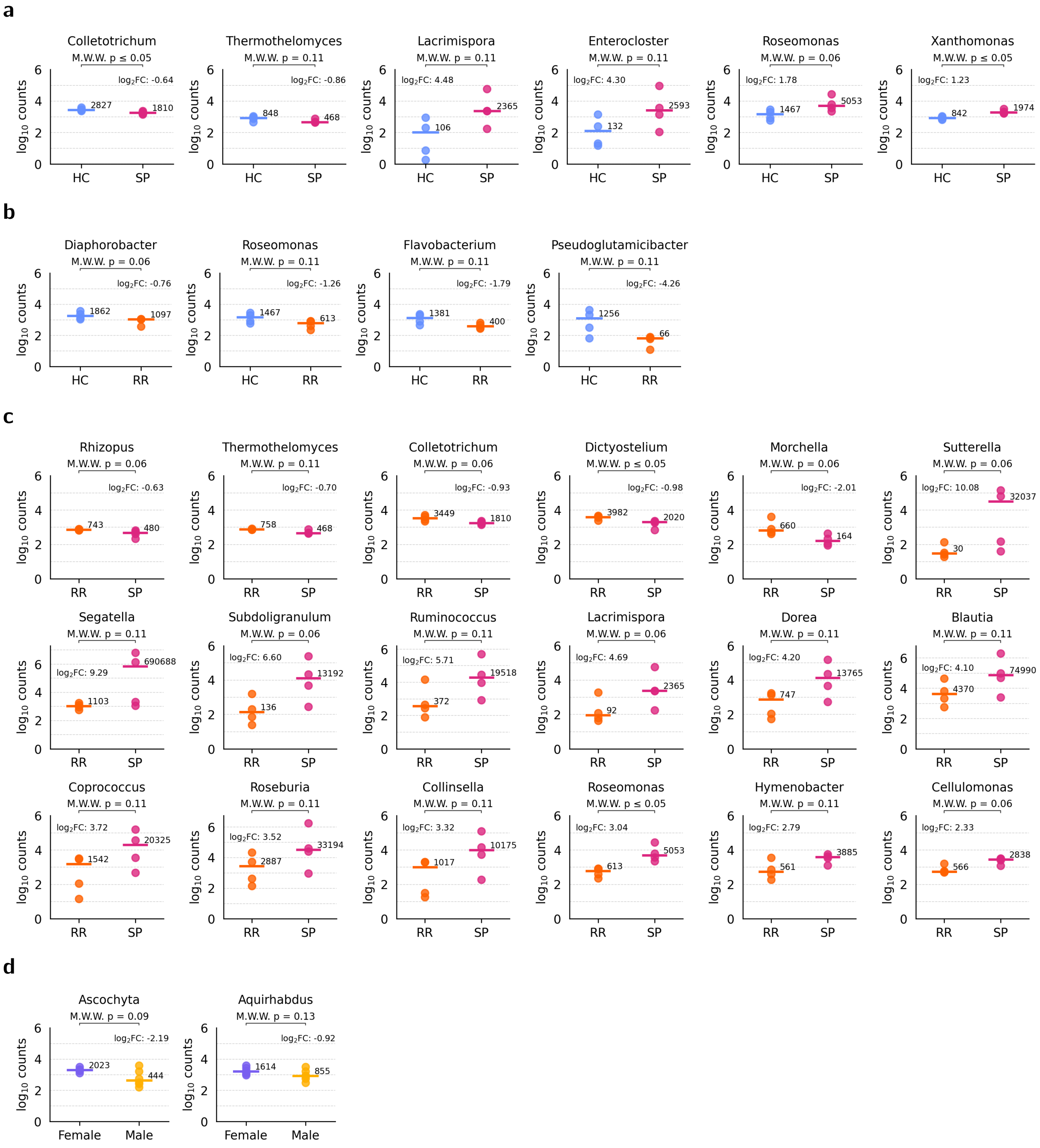

Figure S13: Compared genera across conditions. Reported genera with $p < 0.15$ and the highest $\log_2$FC are shown for the following comparisond: HC vs. SP (a), HC vs. RR (b), RR vs. SP (c) and sex (d). Each plot represents $\log_{10}$ transformed counts for each type of sample, with the $\log_{10}$ transformed median value depicted in a horizontal bar.
